# Low temperature synthesis of plasmonic molybdenum nitride nanosheets for surface enhanced Raman scattering

**DOI:** 10.1038/s41467-020-17628-0

**Published:** 2020-08-04

**Authors:** Haomin Guan, Wencai Yi, Tao Li, Yahui Li, Junfang Li, Hua Bai, Guangcheng Xi

**Affiliations:** 1 Institute of Industrial and Consumer Product Safety, Chinese Academy of Inspection and Quarantine, No. 11, Ronghua South Road, Beijing, 100176 P. R. China; 2 School of Physics and Physical Engineering, Qufu Normal University, Qufu, 273165 P. R. China

**Keywords:** Chemistry, Materials science, Nanoscience and technology, Optics and photonics

## Abstract

Molybdenum nitride (δ–MoN) is an important functional material due to its impressive catalytic, energy storage, and superconducting properties. However, the synthesis of δ–MoN usually requires extremely harsh conditions; thus, the insight into δ−MoN is far behind that of oxides and sulfides of molybdenum. Herein, we report that ultrathin δ−MoN nanosheets are prepared at 270 °C and 12 atm. WN, VN, and TiN nanosheets are also synthesized by this method. The δ−MoN nanosheets show strong surface plasmon resonance, high conductivity, excellent thermal and chemical stability as well as a high photothermal conversion efficiency of 61.1%. As a promising surface enhanced Raman scattering substrate, the δ−MoN nanosheets exhibit a 8.16 × 10^6^ enhanced factor and a 10^−10^ level detection limit for polychlorophenol.

## Abstract (web summary)

Molybdenum nitride is promising for catalysis, energy storage and Raman scattering, but it is synthesized under harsh conditions. Here the authors synthesize highly crystalline molybdenum nitride nanosheets using a relatively mild, non-aqueous solvothermal approach that can be extended to other nitrides.

## Introduction

Because of their excellent catalytic activity, high mechanical strength, and excellent conductivity^1–4^, transition metal nitrides have attracted more and more attention in catalysis^5–7^, energy^8,9^, sensing^10–12^, and other applications^13–15^. Because of their importance, researchers have synthesized a variety of transition metal nitride nanostructures, such as mesoporous VN^16^, Ti_2_N nanosheets^17^, CoN nanowires^18^, NiMoN_x_ nanowires^19^, and Ni_3_N nanosheets^20^. Among these transition metal nitrides, δ−MoN has attracted extensive attention due to their catalytic properties similar to platinum-group metals, energy storage, and superconductivity properties^21–24^. However, δ−MoN is inert in thermodynamics under an ambient pressure, so it is a great challenge to prepare δ−MoN with controllable structures^25^. So far, the synthesis of δ−MoN generally requires high temperature (above 1000 °C) and high pressure (up to severe GPa)^26^. For example, Bezinge et al. prepared δ−MoN film under the pressure of 6 GPa and the reaction temperature of 1500 °C^27^. Similarly, Inumaru et al. synthesized δ−MoN crystalline film under the pressure of 10 GPa and the temperature of 1027 °C^28^. Later, with the efforts of Jia et al., highly crystalline δ−MoN superconducting films were successfully prepared by a polymer-assisted deposition at a atmospheric pressure and 900 °C^29^. Recently, a breakthrough has been made in the synthesis of δ−MoN nanostructures. Gogotsi et al. prepared δ−MoN nanosheets and arrayed nanocrystals at 650 °C under an ambient pressure using NaCl as template^30,31^. More recently, Liu and Sun et al. have synthesized mesoporous transition metal nitrides by biological-template nitriding at 650 °C under an ambient pressure^32^. Although continuous progress has been made in the preparation technology, the synthesis conditions of crystalline δ−MoN are still quite harsh, which to some extent limits their research and application.

Through more than 40 years of continuous progress, surface enhanced Raman scattering (SERS) technology has become a powerful analysis tool, and is widely used in hazardous substance testing^33^, cell imaging^34^, reaction process monitoring^35^, and other fields. Among the many factors that determine the performance of SERS, the substrate that can cause enhanced Raman scattering is indeed the most important^36^. Noble-metals and semiconductors are the two most studied types of SERS substrates. For noble-metal substrates that are enhanced by surface plasmon resonance (SPR), although they have high sensitivity, they also have the disadvantages of high cost and poor biocompatibility^37^. For semiconductor substrates that have enhancement effects caused by charge-transport, their advantages are adjustable band gap and high biocompatibility, but their disadvantages are lower sensitivity and poor stability^38^. Therefore, the development of new SERS substrates with high sensitivity, reliable stability, and excellent biocompatibility is very meaningful for the applications of SERS technology.

The above-mentioned methods are basically based on the high temperature gas–solid phase reaction, so far, no low temperature solution method has been reported for the synthesis of transition metal nitride nanosheets. Herein, we report a low temperature (270 °C) and low pressure (12 atm) solution route for the preparation of δ−MoN nanosheets and other transition metal nitride nanosheets for the first time. In this method, the highly crystallized δ−MoN ultrathin nanosheets were successfully prepared by using Li_3_N and MoCl_3_ as nitrogen and molybdenum sources, stable O-xylene as solvent and ethylenediamine (EDA) as structure guide agent. By using this method, crystalline VN, WN, and TiN nanosheets are also synthesized. These prepared δ−MoN nanosheets have high conductivity and thermal stability. Based on their strong localized surface plasmon resonance (LSPR) effect, the δ−MoN nanosheets show excellent photothermal conversion effect and outstanding SERS properties. To the best of our knowledge, this may be the first time to report the photothermal and SERS properties of δ−MoN nanomaterials.

## Results

### Synthesis and characterization of δ−MoN nanosheets

**Fig. 1:**
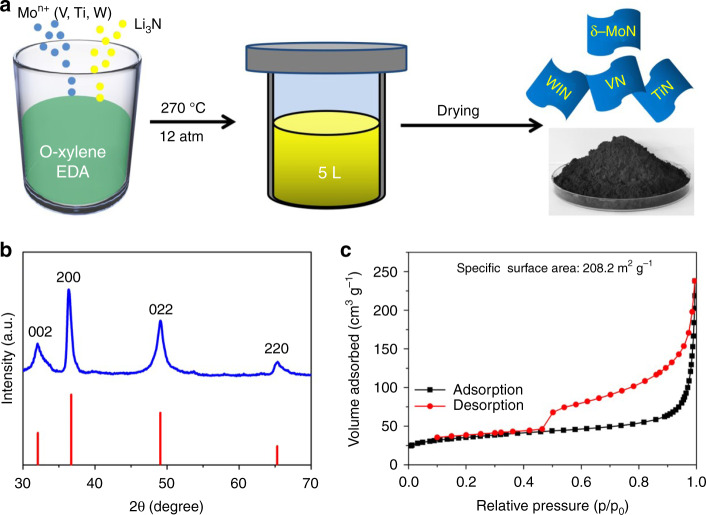
Synthesis and crystal phase of the δ−MoN nanosheets. **a** Schematics illustrating the synthesis of highly crystalline δ−MoN nanosheets. **b** XRD pattern and **c** N_2_ adsorption and desorption isotherms of the δ−MoN nanosheets.

The synthesis scheme of the δ−MoN nanosheets is shown in Fig. 1a. In the synthesis, the chemically active MoCl_3_ and Li_3_N are used as Mo source and N source respectively, which can greatly reduce the reaction barrier for the formation of δ−MoN with high lattice energy. Since both of the two precursors are prone to hydrolysis, o-xylene, which has relatively inert chemical properties, is selected as the reaction solvent. Benzene can also be used as the reaction solvent, but o-xylene is much less toxic than benzene, therefore, so o-xylene is a better choice. On the other hand, in order to obtain two-dimensional (2D) nanostructures, EDA is added to the reaction system as a structure directing agent. After filtrating, washing and freeze-drying, black powder products were obtained.

Firstly, X-ray powder diffraction (XRD) was used to determine the crystal phase (Fig. 1b) of these black products. The characterization results shown that the XRD patterns of the samples are highly consistent with that of the hexagonal δ−MoN (PDF: 01-089-5024). We note that these diffraction peaks are significantly broadened, suggesting that the sample particles are very small in at least one dimension. The Raman spectrum of the sample can also be characterized by δ−MoN (Supplementary Fig. 1). The X-ray photoelectron spectroscopy of these samples confirmed the existence of Mo and N. The peak at 394.9 eV can be regarded as Mo–N bond, while the peak at 397.6 eV can be indexed as N 1 s peak, which proves that δ−MoN is formed^30^. In addition, there is a weak Mo-O peak at 399.2 eV, which can be attributed to the surface hydroxyl, carbonyl, and other groups (Supplementary Fig. 2). Moreover, N_2_ adsorption and desorption experiments revealed that the specific surface area of these δ−MoN samples is quite large, reaching 208.2 m^2^ g^−1^ (Fig. 1c), which can be attributed to the lower reaction temperature hindering the formation of large grains. Such a high specific surface area is very significant for improving the catalytic and sensing properties of the materials.

**Fig. 2:**
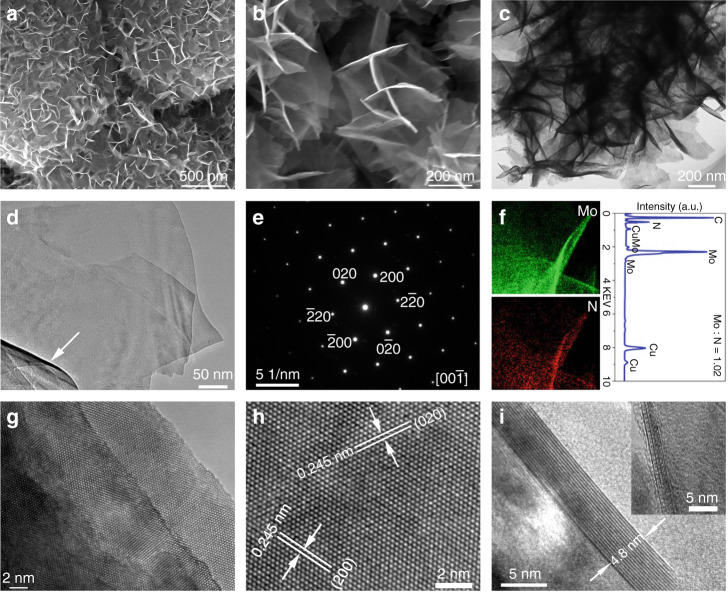
Morphology and structure characterization of the δ−MoN nanosheets. **a**, **b** SEM images. **c**, **d** TEM images. **e** SAED pattern. **f** EDS spectrum and mapping. **g**–**i** HRTEM images.

Then scanning electron microscopy (SEM) and transmission electron microscopy (TEM) were used to characterize the size and morphology of the samples. As shown in Fig. 2a and Supplementary Fig. 3, the low-magnification SEM image show that these samples are honeycomb structures formed by many cross-linking and self-assembly of nanosheets. The high-magnification SEM image (Fig. 2b) further proves that the sample is composed of large number of interconnected nanosheets. It should be noted that the high transparency of the SEM image suggests that these nanosheets are very thin, which is only about 2–5 nm in thickness. Furthermore, atomic force microscopy (AFM) image also proves that the thickness of the δ−MoN nanosheets does not exceed 5 nm (Supplementary Fig. 4). According to the low-magnification TEM (Fig. 2c and Supplementary Fig. 5) and the high-angle annular dark-field (HAADF) images (Supplementary Fig. 6), these samples are full of nanosheets from the outside to the inside. The enlarged TEM image (Fig. 2d) shows that the surface of these nanosheets is highly smooth, and its large curvature proves its remarkable flexibility (marked with arrow). The highly ordered selected area electron diffraction (SAED) pattern revealed the high crystallinity of the δ−MoN nanosheets (Fig. 2e). Energy dispersion spectrum (EDS) mapping demonstrated that the distribution of Mo and N elements in the nanosheets is very uniform (Fig. 2f and Supplementary Fig. 7), and the atomic ratio of Mo and N is about 1.02–1.07. High resolution inductive coupled plasma emission spectrometer (ICP) was used to identify whether Li was doped in the MoN nanosheets. The results showed that no detectable Li signal was detected. High resolution TEM (HRTEM) was used to detect the microstructure of the δ−MoN nanosheets. Figure 2g and Supplementary Fig. 8 clearly shows the growth steps of the nanosheets, which is highly consistent with the previously reported δ−MoN nanosheets prepared by high temperature nitridation method^30^. A large-scale HRTEM image (67 nm × 37 nm) reveals that the crystallinity of the δ−MoN nanosheets is quite high on the whole (Supplementary Fig. 9), and the surface of these nanosheets is very clean, and no attachments is found. As shown in Fig. 2h, the crystal planes with a lattice spacing of 0.245 nm can be characterized as (200) plane, while the other lattice spacing of 0.245 nm can be characterized as (020) plane. Figure 2i shows the HRTEM image of the cross-sections of the nanosheets which shows that the thickness of the nanosheet is only 4.8 nm, and no oxide layer or amorphous layer is found. HRTEM image also demonstrated that even thinner nanosheest with thickness less than 2 nm also have excellent crystallinity (inset in Fig. 2i). These characterization results show that highly crystalline ultrathin δ−MoN nanosheets with exposed (001) plane have been successfully prepared by this facile low temperature method.

### Formation mechanism of the δ-MoN nanosheets

**Fig. 3:**
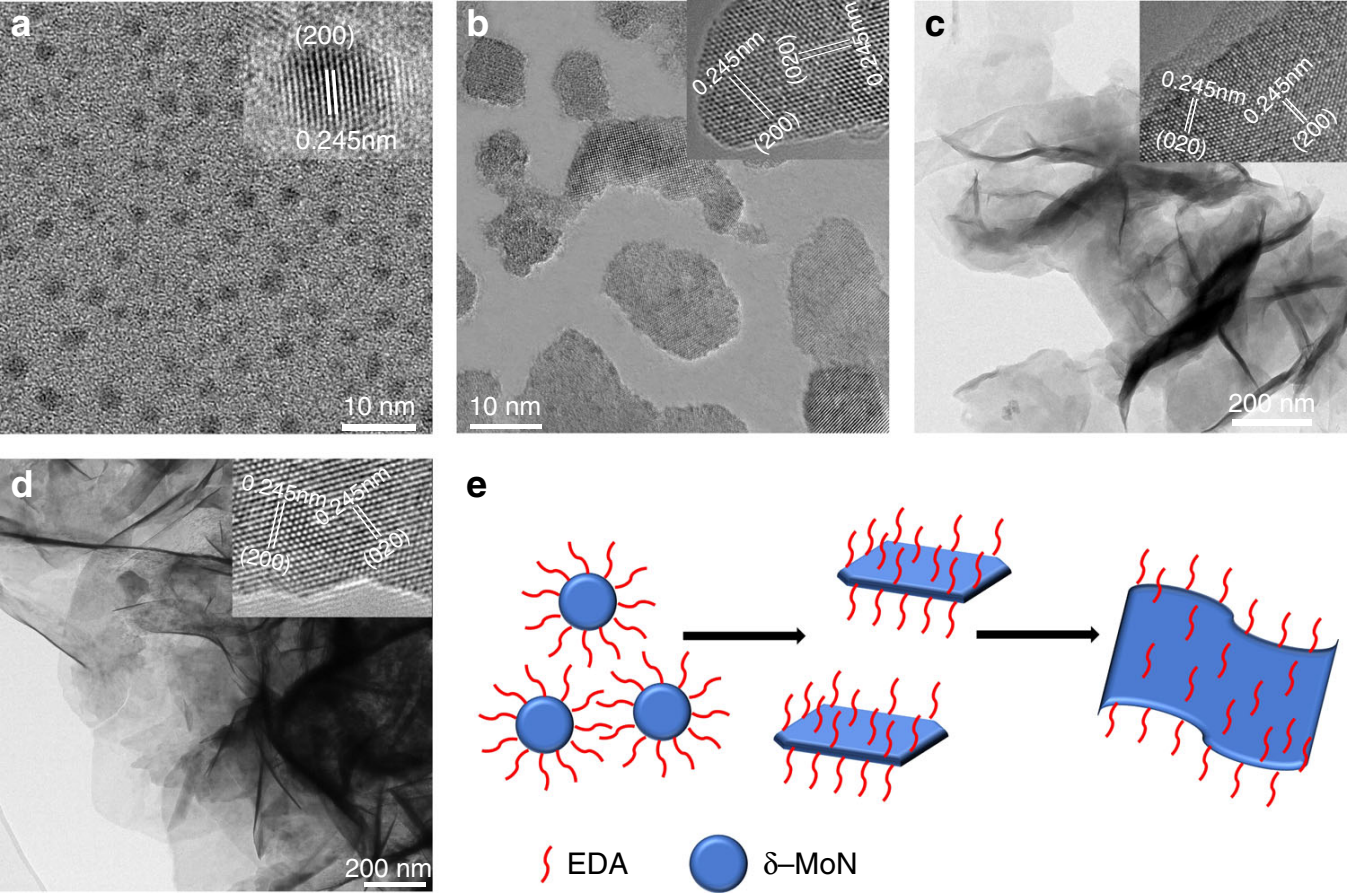
Growth process and formation mechanism of the δ−MoN nanosheets. **a** Quantum dots formed after 1 h reaction, inset: HRTEM image of one quantum dots. **b** 2D nanostructures formed after 3 h reaction, inset: HRTEM image of the 2D nanostructures. **c** Nanosheets formed after 6 h reaction, inset: HRTEM image of the growth step. **d** δ−MoN nanosheets formed after 12 h reaction, inset: HRTEM image of the nanosheets. **e** The proposed Formation mechanism of the δ−MoN nanosheets.

Because this may be the first time to prepare crystalline δ-MoN nanosheets by solution method, it is necessary to explore their formation mechanism. In order to gain insight into the formation mechanism of the δ−MoN nanosheets, we investigated their growth process in detail. TEM image show that the obtained product after 1 h reaction is δ−MoN quantum dots of 2–3 nm (Fig. 3a). After 3 h reaction, the quantum dots grow to form small nanoplates (Fig. 3b). By prolonging the reaction time to 6 h, these small nanoplates developed into larger nanosheets (Fig. 3c). Finally, when the reaction time increased to 12 h, a large number of nanosheets were produced (Fig. 3d). This process of morphology evolution proves that the formation of the δ−MoN nanosheets follows the classic nucleation-growth process, and no Ostwald ripening and oriented attachment phenomena were found. It is worth noting that we have found obvious growth steps on the newly formed nanosheets (inset in Fig. 3c), which is the direct evidence of the nucleation-growth mechanism.

Comparative experiments show that if no EDA is added to the reaction system, no δ−MoN nanosheets are formed. TEM and HRTEM images show that in the absence of EDA, although crystalline δ−MoN nanoparticles can still be synthesized, their morphology is irregular (Supplementary Fig. 10). The results demonstrated that EDA played an important role in the formation of the ultrathin δ−MoN nanosheets. As an important and common bidentate-ligand molecule, it is well known that EDA is an organic molecule with strong coordination ability. It has been found to play an important role as a structural directing agent in the formation of metal sulfide and oxide nanostructures^39–41^. In the current case, we notice that from nanoplates to nanosheets, their exposed crystal faces are all (001) planes (see HRTEM images inset in Fig. 3b–d). These evidence suggest that EDA can be preferentially adsorbed on the (001) crystal plane of δ−MoN in the solution. In-situ high-resolution mass spectrometry was used to prove this hypothesis. The results show that the EDA molecules and Mo ions combine to form a Mo-EDA complex on the surface of the δ−MoN nanosheets (Supplementary Fig. 11), which clearly proves the crystal plane adsorption effect of EDA molecule. To prove this more fully, Raman spectroscopy was used to detect the distribution of EDA molecules on the surface of the δ−MoN nanosheets. As shown in Supplementary Fig. 12, the Raman scattering signal of EDA is very uniformly distributed on the surface of the nanosheets, which further proves the adsorption of EDA molecules. It should be noted that these adsorbed EDA molecules can be washed away in a suitable solvent. FTIR spectrum showed that no EDA signal was detected on the surface of MoN nanosheets after being washed with DMF and ethanol (Supplementary Fig. 13). At the same time, the Raman spectrum detection of the MoN nanosheets shows that no Raman signal of EDA appears, only the Raman signal of MoN itself is detected (Supplementary Fig. 1). On the other hand, increasing the reaction temperature to 350 °C will lead to the formation of a mixture of δ−MoN nanoparticles and nanosheets even in the presence of EDA (Supplementary Fig. 14), which can be attributed to the increased EDA desorption effect caused by the increase in temperature. These results demonstrated that the morphology of the δ−MoN nanocrystals can be precisely controlled with the appropriate complexing agents. The proposed formation mechanism is summarized in Fig. 3e.

**Fig. 4:**
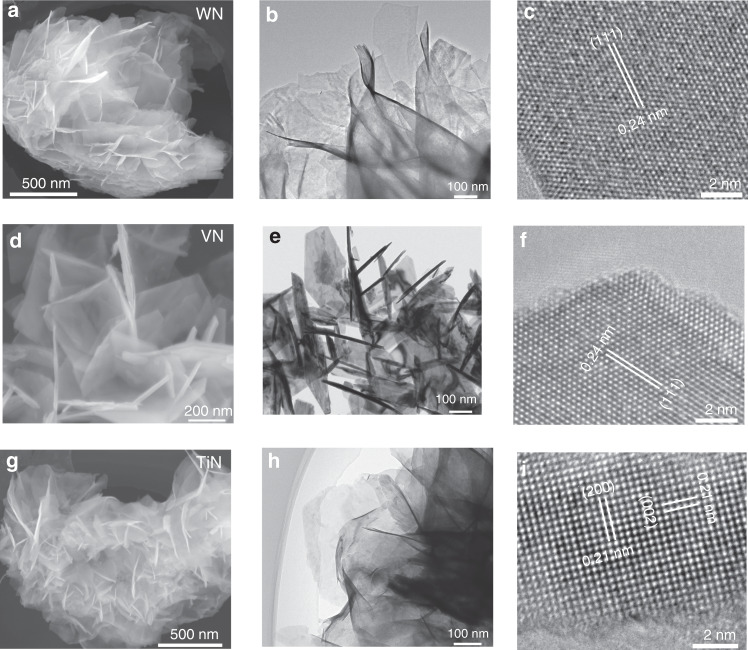
Morphology and structure characterization of the nanosheets. **a**–**c** WN nanosheets. **d**–**f** VN nanosheets. **g**–**i** TiN nanosheets.

This low temperature solution method is not only suitable for synthesizing δ−MoN nanosheets, but also for synthesizing other typical transition metal nitride nanosheets. Figure 4 show the SEM, TEM, and HRTEM images of the synthesized WN, VN, and TiN nanosheets. SEM and TEM images show that all the samples are in the form of nanosheets, and the thickness of them is about 2–5 nm. The HRTEM images (Fig. 4c, f, i) reveal the typical lattice fringe spacing of 0.24, 0.24, and 0.21 nm, corresponding to WN (111), VN (111) and TiN (200) crystal faces respectively. Moreover, the successful synthesis of different types of transition metal nitrides is also confirmed by their XRD patterns shown in Supplementary Fig. 15. All diffraction peaks of the three samples can be characterized as WN (JCPDS No. 65-2898), VN (JCPDS No. 35-0768), and TiN (JCPDS No. 38-1420). These exhaustive characterization results convincingly prove that this EDA assisted low temperature solution method is an effective and general way to synthesize transition metal nitride nanosheets.

### Thermal and chemical stability of the δ−MoN nanosheets

**Fig. 5:**
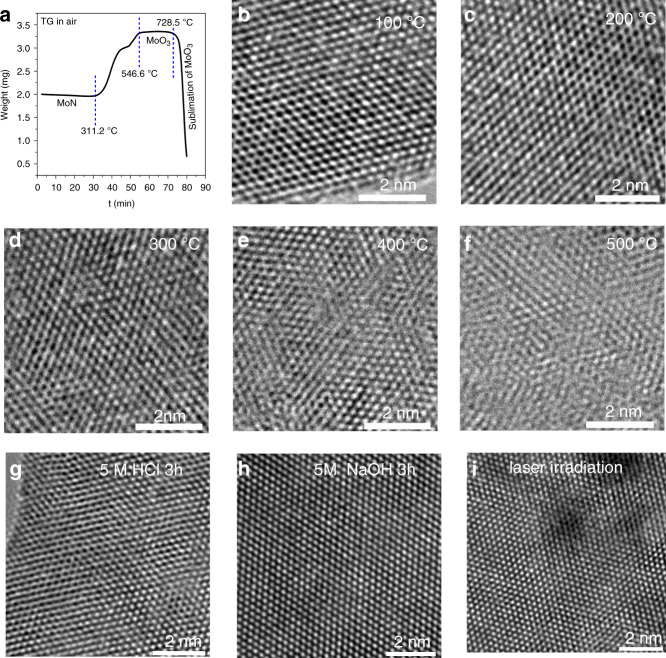
Thermal, chemical, and light stability of the δ−MoN nanosheets. **a** TG curve. **b**–**f** HRTEM images recorded from the nanosheets treated at different temperatures. **g**–**i** HRTEM images of the δ−MoN nanosheets treated with HCl, NaOH, and laser irradiation.

It is well known that the bulk δ−MoN is probably one of the most stable materials, with extremely high hardness, melting point, and corrosion resistance^25,27^. In order to test whether these ultrathin δ−MoN nanosheets still have these excellent properties, we examined their thermal and chemical stability. First of all, the thermogravimetry (TG) curve showed that there was no significant weight change in these nanosheets before 311.2 °C (Fig. 5a). Beyond this temperature, these δ−MoN nanosheets are gradually oxidized to MoO_3_. At 546.6 °C, these MoN are completely converted into MoO_3_. When the temperature is over 728.5 °C, the MoO_3_ sublimates. On the other hand, with the increase of temperature, HRTEM was used to characterize the change of crystal structure of these nanosheets. From these images (Fig. 5b–d), it can be seen that below 300 °C, these nanosheets basically maintain high lattice order. With the increase of temperature to 400–500 °C, a large number of amorphous layers and lattice defects (Fig. 5e, f) appeared on the nanosheets, indicating that the lattice of δ−MoN was destroyed and amorphous molybdenum oxide was formed. These TG data and HRTEM images results demonstrate that these δ−MoN nanosheets have excellent thermal stability and oxidation resistance. In addition, these δ−MoN nanosheets also show extremely excellent chemical stability and can withstand the corrosion of strong acids and alkalis. Figure 5g–h give the HRTEM images of the δ−MoN nanosheets treated with HCl (5 M, 3 h) and NaOH (5 M, 3 h), showing that the nanosheets still show high crystallinity after severe corrosion treatment. Considering that the substrate material is exposed to the laser (excitation light) for a long time (2–5 h) when doing SERS experiments, the photostability of the nanosheets has also been investigated. As shown in Fig. 5i, the results show that there is no appreciable change in the surface structure of the nanosheets after 3 h of laser irradiation (633 nm, 20 mW) with a spot diameter of 700 μm. The high thermal and chemical stability are also demonstrated by their almost unchanged XRD patterns (Supplementary Fig. 16) and XPS spectra (Supplementary Fig. 17). Thus the exhaustive characterized results convincingly prove that these ultrathin and highly crystalline δ−MoN nanosheets have excellent thermal, chemical, and light stability.

### LSPR effect and superhydrophobicity of the δ−MoN nanosheets

**Fig. 6:**
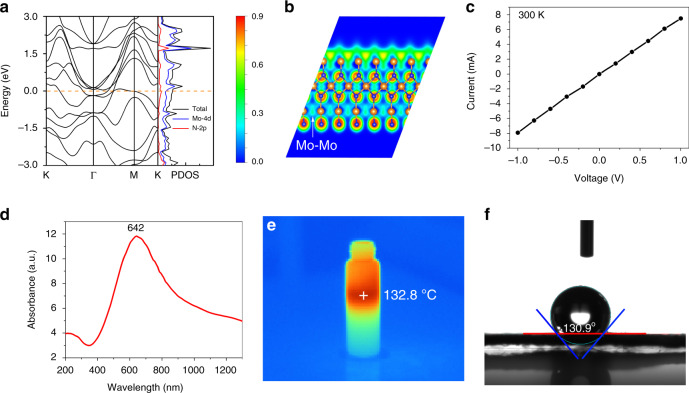
Characterization of the physical properties of δ−MoN nanosheets. **a**, **b** DOS and EIF of δ−MoN. **c** I– V curves of the δ−MoN nanosheets at 300 K. **d** UV-Vis absorption of the δ−MoN nanosheets. **e** Photothermal effect of the δ−MoN nanosheets. **f** Contact angle measured at the interface of water drop and nanosheets.

Theoretical calculations show that there is no band gap in δ−MoN, combined with the high d-orbital electron density near the Fermi level (Fig. 6a). Besides, the free electron density distribution, which was probed by calculating the electron localization functions (ELF), indicates that the free electron gas density of δ−MoN is quite high, and forms large amount of Mo–Mo metallic bonds (Fig. 6b), indicating that δ−MoN is distinct metallic. To probe the conductivity of the highly crystalline δ−MoN nanosheets, the current-voltage curve was measured. The results display that the δ−MoN nanosheets have good conductivity at 300 K, and the linear temperature dependence of conductivity are highly consistent with metals (Fig. 6c). The obtained resistivity value is only ∼1.29 × 10^−4^ Ω cm at 300 K, which is comparable to the value of non-metallic nanomaterials with high conductivity (Supplementary Table 1). The δ−MoN nanosheets exhibit a strong LSPR absorption from visible to near infrared regions, and the center of the absorption is at 642 nm (Fig. 6d). In contrast, commercial δ−MoN powder (micron scale irregular particles) only exhibits weak absorption in the visible region (Supplementary Fig. 18). The reason for this different optical phenomenon may be because the nanosheet has a larger light-absorbing surface, a thinner thickness, and a large number of nanogap (easy to form electromagnetic field hot spots). It should be noted that this may be the first time that such a strong LSPR effect has been found over the δ−MoN nanostructures. Moreover, due to the strong LSPR effect, the δ−MoN nanosheets exhibit excellent photothermal effects. Under the irradiation of simulated sunlight (xeon light, 2 KW m^−2^), the local temperature of the dispersion of these δ−MoN nanosheets (20 mL, 10 mg 100 mL^−1^) reached a maximum of 132.8 °C within 20 s (Fig. 6e). The photothermal conversion efficiency of δ−MoN nanosheets was calculated according to the reported method (see Supporting Information and Supplementary Fig. 19)^42^, and the efficiency of these MoN nanosheets irradiated by 633 nm laser is counted at 61.1%, which is excellent even compared with the noble metal nanomaterials^43^. In addition, the wettability of the δ−MoN nanosheets was test with contact angle measurement (Fig. 6f). It is observed that the contact angle of water against the δ−MoN nanosheets to be about 130.9°, which proves that the surface of these nanosheets is superhydrophobic.

### Enhanced Raman scattering properties

**Fig. 7:**
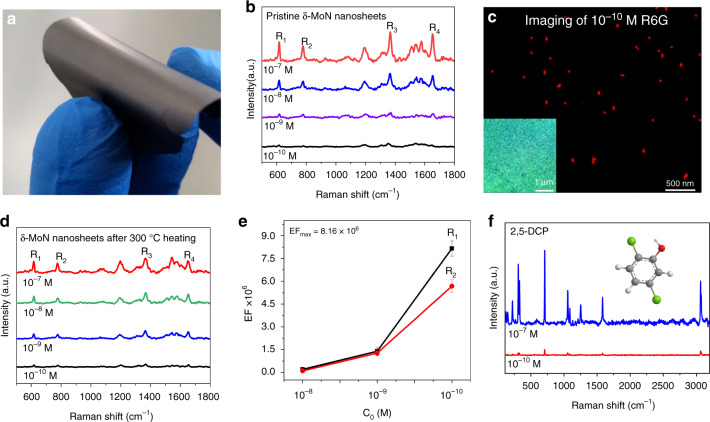
SERS properties of the δ−MoN nanosheets. **a** Flexible SERS substrate assembled with the δ−MoN nanosheets. **b** Raman spectra of 10^−7^–10^−10^ M R6G obtained in the flexible δ−MoN substrate. **c** SERS imaging recorded from 10^−10^ M R6G over the δ−MoN substrate, inset: an optical photo of the selected area. **d** Raman spectra of R6G recorded on the δ−MoN substrate after 300 °C heat treatment. **e** The average Raman EFs obtained by counting the peak intensities (R_1_ and R_2_) at three different concentration levels. **f** Raman spectra of 2,5-DCP obtained in the flexible δ−MoN nanosheet substrate.

The superconducting and catalytic properties of δ−MoN are well known, but other properties are rarely reported. Strong LSPR often makes the materials have good SERS properties according to the electromagnetic field enhancement mechanism (EM) of Raman scattering^33,44^. Therefore, we systematically evaluated the SERS properties of these δ−MoN nanosheets. All of these Raman tests are carried out on the flexible film formed by the δ−MoN nanosheets (Fig. 7a). The flexible films are obtained by a facile suction-filtration method (details see “Methods”). If there are no special instructions, the excitation wavelength is 633 nm in all Raman experiments and the power is 0.5 mW. When the concentration of 10^−7^ M R6G is used as probe molecules, the enhanced Raman signals of R6G can be clearly detected on the δ−MoN film, and four characteristic peaks appear (Fig. 7b). Through the detection of a series of R6G solutions with different concentrations (10^−7^–10^−10^ M), as a high active SERS substrate, the lowest detection limit (LOD) of the δ−MoN nanosheets for R6G is 10^−10^ M (S/N ≥ 5), which is comparable to that of noble-metal substrates. Furthermore, these δ−MoN nanosheets also show excellent SERS imaging performance. As shown in Fig. 7c, the size of these red imaging spots is between 3 and 13 nm. The size of a single R6G molecule is about 1.5–2 nm, therefore, it can be concluded that these bright spots with different sizes represent the imaging from double molecules to several molecular aggregate.

In addition to the unexpected high sensitivity, the δ−MoN nanosheet substrate exhibits excellent stability and reliability. Experimental results have revealed that the δ−MoN nanosheets still maintain the 10^−10^ M detection limit to R6G (Fig. 7d), even if heated at 300 °C for 1 h. In addition, 10^−10^ M LOD can also be maintained in the δ−MoN nanosheets treated with strong acid and strong alkalior (Supplementary Fig. 20). Such high stability means that the δ−MoN nanosheets have a promising application prospect in the actual SERS detection.

As the most important evaluation index of substrate performance, the calculated maximum Raman EF based on R_1_ peak is up to 8.16 × 10^6^ (Fig. 7e). As far as we know, this value is higher than that of most semiconductor substrates, and even comparable to noble metal substrates. Such excellent SERS performance can be rationally attributed to the strong LSPR effect, large specific surface area, and high crystallinity of the δ−MoN nanosheets, as well as a large number of gaps and pores among the nanosheets that easily generate electromagnetic hot spots.

For practical SERS detection, the reproducibility of Raman signal obtained on the substrate is also an important indicator. In order to achieve high reproducibility, R6G (1 × 10^−7^ M) was used as the probe to estimate the repeatability of the MoN-based substrate. First, 100 measuring points were selected randomly from the MoN substrate (Supplementary Fig. 21a shows the optical photo), and the spectra show that the signal values are highly consistent (Supplementary Fig. 21b). In order to further inspect the high signal repeatability, the Raman spectra obtained from 5000 measuring points from the area shown in Supplementary Fig. 21a were used to calculate their relative standard deviation (RSD). The mapping images of R_1_ and R_2_ scattering peaks obtained by scanning 5000 measurement points shows a highly consistent signal response, which indicates that the probe molecules are evenly distributed on these MoN-nanosheet substrate (Supplementary Fig. 21c, d). By calculating the peak intensity distribution of R_1_ and R_2_ in these 5000 Raman spectra, the RSDs obtained are 6.9% and 7.8%, respectively (Supplementary Fig. 21e, f). Such low RSD undoubtedly proves the high signal repeatability of the MoN-nanosheet substrate.

It should be noted that for some substances that are not easily soluble in water, such as dichlorophenol, trichlorophenol, and other oil soluble environmental pollutants, it is difficult to detect them with the surface hydrophilic noble-metal substrates, unless their surface is modified with particular molecules. Fortunately, these surface superhydrophobic δ−MoN nanosheets can detect these oil-soluble harmful substances sensitively. As shown in Fig. 7f, by using the superhydrophobic δ−MoN nanosheets as SERS substrate, the LOD of 2,5-dichlorophenol (2,5-DCP, a widely concerned environmental pollutant) can reach 10^−10^ M. When the substance to be tested is changed into bisphenol A (BPA, an environmental hormone that easily causes cancer and endocrine disorders), its LOD can still be kept at 10^−10^ M level (Supplementary Fig. 22). These results demonstrate that the superhydrophobic SERS substrate can not only detect trace amount of oil-soluble environmental pollutants, but also prove the universality of the δ−MoN nanosheet substrate.

## Discussion

In summary, a simple low temperature solution method has been developed for the preparation of a series of transition metal nitride nanosheets, such as δ−MoN, WN, VN, and TiN nanosheets. Compared with the common high temperature gas–solid phase synthetic method, the reaction temperature of this method is only 270 °C, which greatly reduces the energy consumption. The obtained δ−MoN nanosheets have high crystallinity, excellent conductivity, large specific surface area, and high thermal stability. These δ−MoN nanosheets show strong LSPR effect and excellent photothermal conversion efficiency. Moreover, the flexible SERS substrate assembled by the δ−MoN ultrathin nanosheets shows the comparable sensitivity and enhancement factor as that of noble-metals. Combined with their low cost and ultra-high stability, these molybdenum nitride nanosheets show extremely promising application prospects. It is believed that this low temperature solution method can be extended to synthesize other 2D metal nitride nanomaterials through appropriate adjustment.

## Methods

### Synthesis of δ−MoN nanosheets

All chemicals used in the experiments are of analytical purity. In a typical synthesis, 1 mmol of MoCl_3_ and 1 mmol Li_3_N were added into a mixed solution of o-xylene (40 mL) and EDA (5 mL), and gently stirred for 2 h at room temperature in a nitrogen protected glove box. Then, the resulting mixture is then sealed in a 80 mL Teflon-lined autoclave and heated to 270 °C at a rate of 3 °C min^−1^ for 20 h. After the reaction is completed, the black products were separated and collected by centrifugation. Finally, the black powders were washed with ethanol, N,N-dimethylformamide (DMF), and distilled water for three times and freeze-dried. VN nanosheets were prepared by the same method as δ−MoN nanosheets.

### Constructing flexible δ−MoN nanosheet SERS substrate

In a typical preparation process, 0.15 g of δ−MoN nanosheets are mixed with 50 mL of distilled water to form a suspension. These suspensions are filtered to form a thin layer on the filter paper. After natural drying in air at room temperature, the flexible MoN film is spontaneously separated from the filter paper to form a flexible SERS substrate.

### SERS tests

To study the SERS properties of these MoN nanosheets, a confocal micro Raman spectrometer (Renishaw-inVia Reflex) is used as the measuring instrument. In all SERS tests, unless specifically stated, the excitation wavelength is 633 nm, laser power is 0.5 mW and the specification of the objective is ×100 L. A series of standard solution of R6G with concentrations of 10^−7^–10^−10^ M were used as the probe molecules. The solution is a mixture of water and ethanol and the volume ratio of water and ethanol is 4 to 1. To improve the signal reproducibility and uniformity, the glass sheet covered with MoN nanosheet films were immersed into a probe solution to be measured for 10 min, then taken out and dried in air for 5 min. In all SERS tests, the laser beam is perpendicular to the top of the sample to be tested with a resultant beam spot diameter of 5 μm. The calculation of EF are provided in Supplementary Methods. The fluorescent background of the probe molecule is deducted by the software that comes with the instrument. As for the Raman imaging, The MoN nanosheet films were immersed in 10 M R6G solution for 5 minutes, and then the films were placed on the observation platform of the Raman spectrometer. The laser wavelength is 633 nm and the beam spot diameter is 0.7 μm. Scan the signals of the R_1_ peak at 10,000 points, and convert these signals into the image mode through the software provided with the instrument. When the probe molecules are 2,5-DCP and BPA, the solvent is absolute ethanol, and other experimental details are the same as R6G.

### Characterization

These samples were measured by a variety of characterization techniques. XRD patterns of the products were obtained on a Bruker D8 focus X-ray diffractometer by using CuKa radiation (l = 1.54178 Å). SEM images and EDS were obtained on a Hitachi S-4800. TEM and HRTEM characterizations were performed with a Tecnai G F30 operated at 300 kV. Ultraviolet–Vis absorption spectra were recorded with a Shimadzu UV3600. X-ray Photoelectron Spectroscopy (XPS) experiments were performed in a ESCALab250Xi using monochromated Al Kα X-rays at hυ = 1486.6 eV. Peak positions were internally referenced to the C1s peak at 284.6 eV. The Fourier transform infrared spectra were measured from THERMO Iz-10. The specific surface area was measured in a Micro Tristar II 3020. Raman spectra were recorded from Renishaw-inVia Qontor. High resolution mass spectrometry were obtained from a Thermo-Q Exactive Focus (NanoESI, m/z 50−500).

### Electronic structure calculations

The details of all density functional theory calculations and EIF simulation are provided in Supplementary Methods.

### Photothermal conversion efficiency

The calculation details of photothermal conversion efficiency of the δ−MoN Nanosheets are provided in Supplementary Information.

## Supplementary information

Supplementary Information

Peer Review File

## Data Availability

The data that support the findings of this study are available from the corresponding author upon request. All reported data are included in the paper and Supplementary materials. The Source Data can be downloaded from: https://pan.baidu.com/s/17YxueceoHnmGjTDgQMjQKg (Code: kmjh).
