# Supplementary material for: Low temperature synthesis of plasmonic molybdenum nitride nanosheets for surface enhanced Raman scattering
Source: Nat Commun. 2020 Aug 4;11:3889. doi: 10.1038/s41467-020-17628-0 (PMC7403429; doi:10.1038/s41467-020-17628-0)
Supplement: Supplementary file 1 — Supplementary Information [file 41467_2020_17628_MOESM1_ESM.pdf]

Low temperature synthesis of plasmonic molybdenum nitride nanosheets for surface enhanced Raman scattering

Guan et al.

Supplementary Methods

Enhanced Factor Calculation

To calculate the EF of the MoN nanosheets, the ratio of SERS to normal Raman spectra (NRS) of R6G was determined by using the following calculating formula:[1]

$$EF = (I_{SERS}/N_{SERS}) / (I_{NRS}/N_{NRS})$$

where I_{SERS} and I_{NRS} refer to the peak intensities of the SERS and NRS, respectively. N_{SERS} and N_{NRS} refer to the number of R6G molecules in the SERS substrate and normal Raman sample, respectively. Laser spot size is about 0.56 μm, depth of laser penetration is about 21 μm,[2] and the density of R6G solid is about 1.15 g cm^{-3}, N_{NRS} was estimated as 5×10^{10}. N_{SERS} is determined by laser spot illuminating on the sample and density of R6G molecule adsorbed on the MoN nanosheet surface (about 0.5 nM cm^{-2}), N_{SERS} is concluded as 2.64×10^{6}. In the SERS measurements, two Raman scattering peaks, R_1 at 612 cm^{-1} and R_2 at 773 cm^{-1} were selected for the calculations of the EF. For comparison, the peak intensities of the bulk R6G directly placed on bare glass slide were detected as NRS data. The intensity was obtained by making average 20 laser spots measurements. Substituting these values of above variable into the equation, the optimum EF could be concluded to be about 8.16×10^{6}.

Calculation of Photothermal Conversion Efficiency. According to the Beer-Lambert Law, the mass extinction coefficient α of the MoN nanosheets can be calculated using equation (1):

$$A = \alpha LC \tag{1}$$

In equation (1), A refers to the absorbance of MoN nanosheets at 633 nm, α is the mass extinction coefficient of MoN nanosheets (L g^{-1} cm^{-1}), L is the optical length of the quartz cuvette (cm), and C is the mass concentration (g L^{-1}). According to Figure S19a, the calculated mass extinction coefficient of MoN is 0.851 L g^{-1} cm^{-1}.

The photothermal conversion efficiency (η) of the MoN nanosheets was determined by equation (2). The temperature change of the MoN nanosheet aqueous solution (1 mg mL^{-1}) was recorded as a function of time under continuous irradiation of 633 nm laser at a power of 1 W cm^{-2}, in which the irradiation lasted for 600 s, and then the laser was shut off (Supplementary Figure 19a).

$$\eta = \frac{\mathrm{h}S(T_{\max} - T_{sur}) - Q_{Dis}}{I(1 - 10^{-A633})} \quad (2)$$

Following the previous reports, the value of hS can be calculated by equation (3) — (5). The T, T_{max} and T_{sur} are random temperature, the maximum temperature after irradiation and the surrounding temperature. In this work, we measured the temperature change at concentration of 1 mg/mL, the T_{max}—T_{sur} = 50.7 °C, and the τ_s was calculated to be 363.25 s by equation (3) and (4) as Supplementary Figure 19b and Supplementary Figure 19c. m and C_p in equation (5) are the mass and heat capacity of solvent (water). In this work m = 1 g, C = 4.2 J g^{-1} °C. The hS value was determined to be 8.79 mW / °C.

$$\theta = \frac{T - T_{sur}}{T_{\max} - T_{sur}} \quad (3)$$

$$\mathrm{t} = \tau_s \ln\theta \quad (4)$$

$$\mathrm{h}S = \frac{\sum_i miC_p, i}{\tau_s} \quad (5)$$

Take the hS value into equation (2). At concentration of 1 mg/mL MoN nanosheets, A_{633}=0.851. Q_{Dis} expresses the heat dissipated due to light absorption by the pure water without MoN nanosheets. It is measured to be 13.45 mW. I is the incident laser power (1000 mW). Substituting these values into equation (2), the 633 nm laser photothermal conversion efficiency (η) of MoN nanosheets can be calculated to be 61.1 %.

DFT Computational Details

All the Density functional theory (DFT) calculations were performed by using the Vienna ab initio simulation package (VASP).[1-2] The exchange-correlation functional was described by generalized gradient approximation of Perdew-Burke-Ernzerhof (GGA-PBE)[3-4] and an energy cutoff of 400 eV for the plane-wave basis was set to expand the one-electron wave function, and Brillouin zone (BZ) integrations were performed using a Monkhorst-Pack theme[5] with k-point mesh of 8 × 8 × 1. Geometry optimizations were stopped until the forces on each atom were less than 0.01 eV/Å.

Supplementary Figures

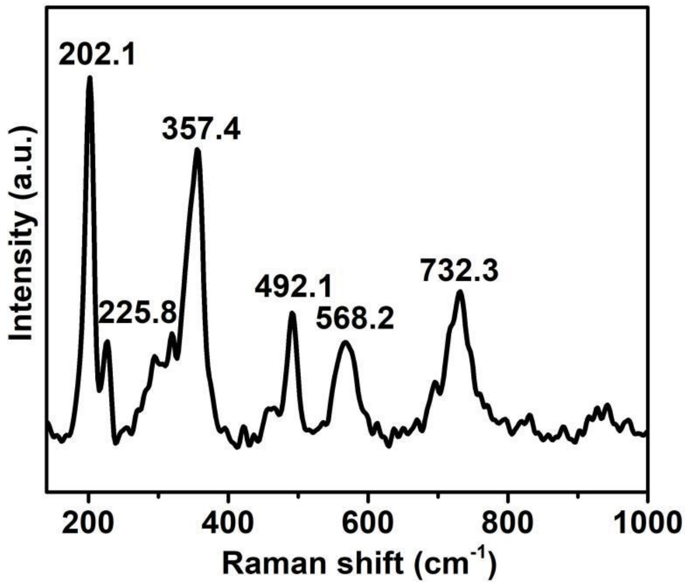

Supplementary Figure 1. A typical Raman spectrum of the as-synthesized δ−MoN nanosheets.

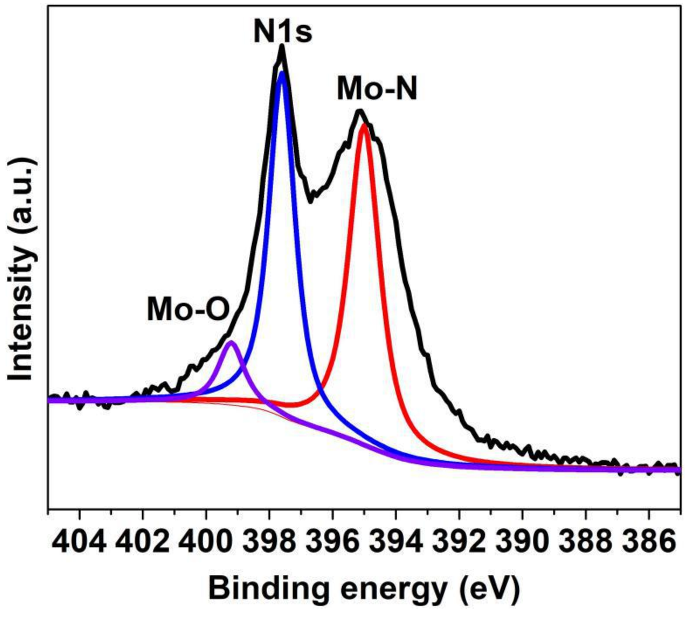

Supplementary Figure 2. N 1s and Mo 3p XPS spectrum of the as-synthesized δ−MoN nanosheets.

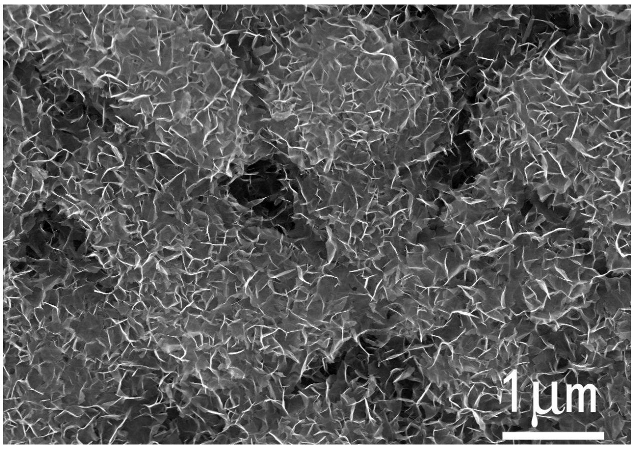

Supplementary Figure 3. Low-magnification SEM image of the honeycomb-like δ–MoN nanosheets.

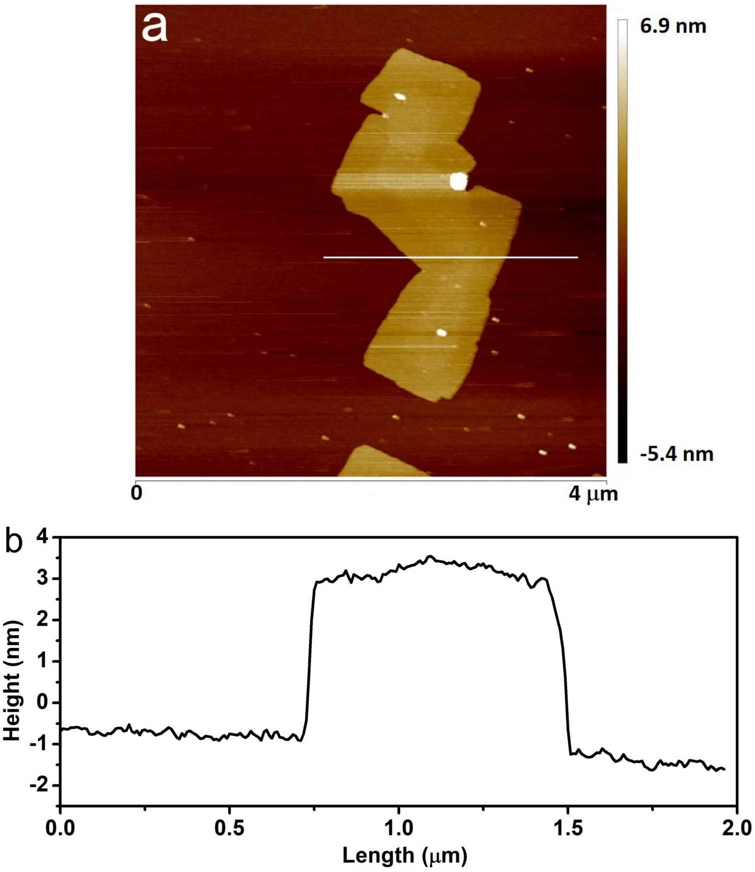

Supplementary Figure 4. The thicknesses of δ-MoN nanosheets was determined using AFM. (a) AFM images of the δ–MoN nanosheets deposited on Si. (b) AFM height profiles measured along the white line in (a).

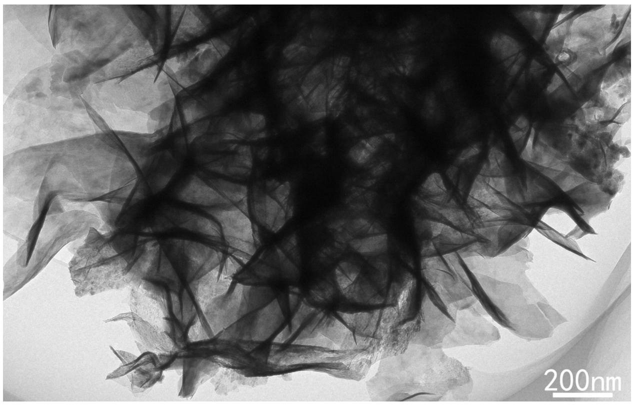

Supplementary Figure 5. Low-magnification TEM image of the δ−MoN nanosheets.

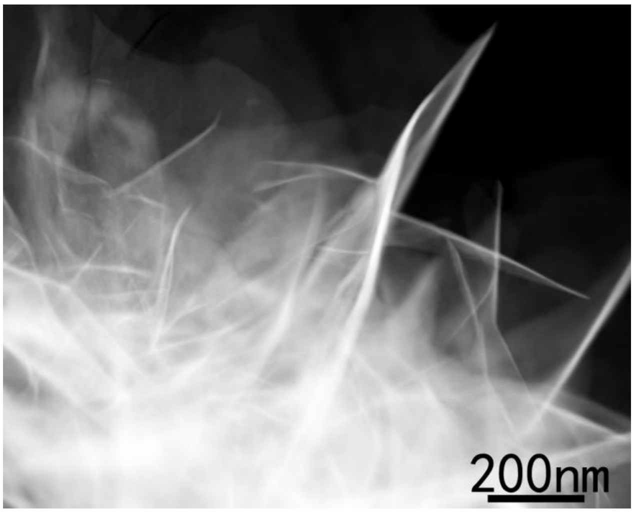

Supplementary Figure 6. HAADF image of the δ−MoN nanosheets.

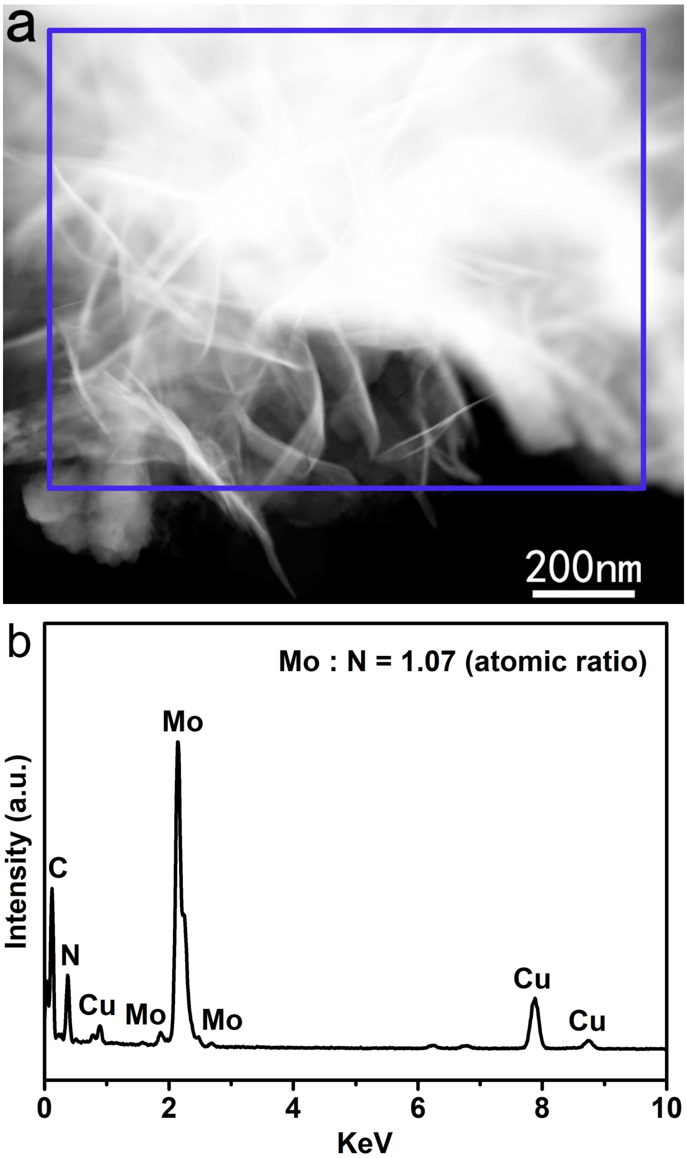

Supplementary Figure 7. EDS spectrum obtained in a large area MoN nanosheets.

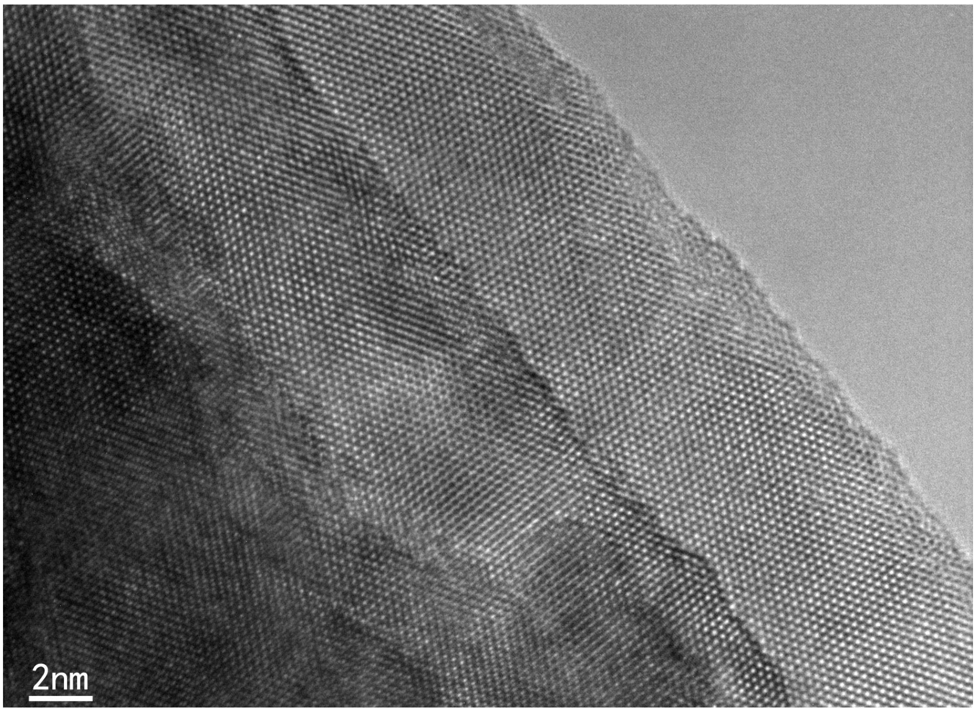

Supplementary Figure 8. Growth steps of the as-synthesized δ–MoN nanosheets.

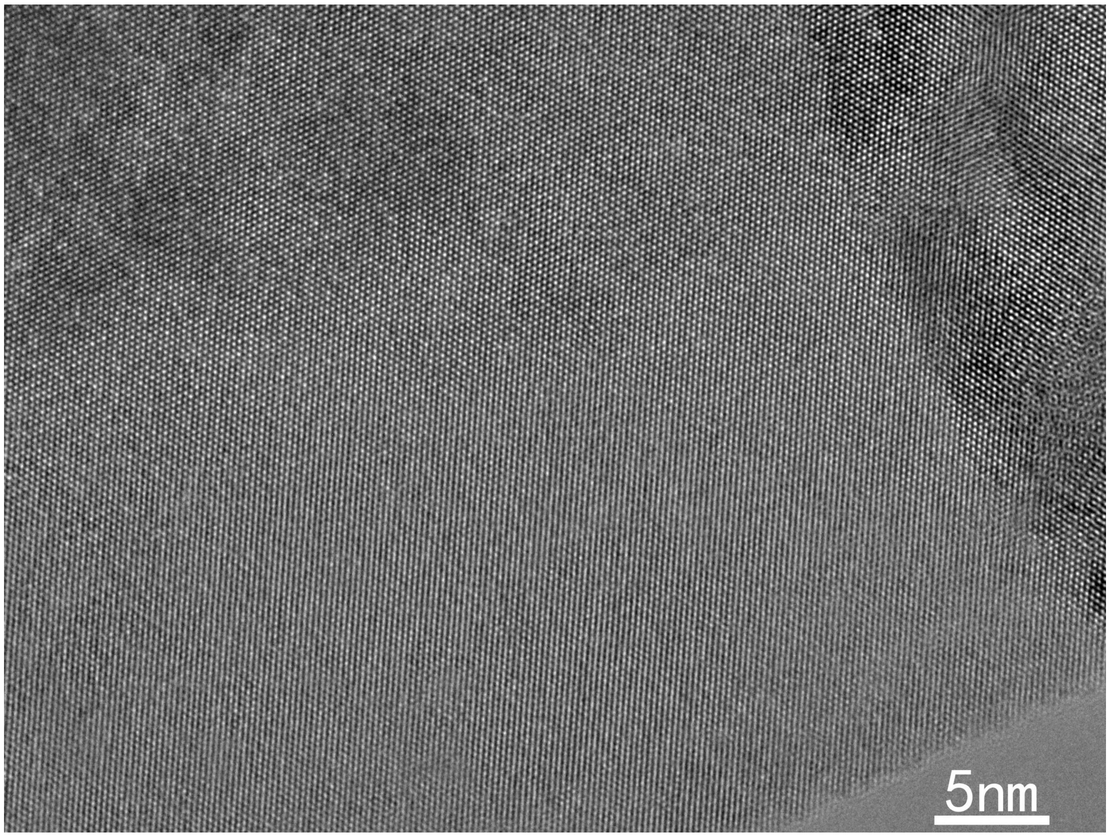

Supplementary Figure 9. Large-sized HRTEM image of the δ–MoN nanosheets, revealing their high crystallinity.

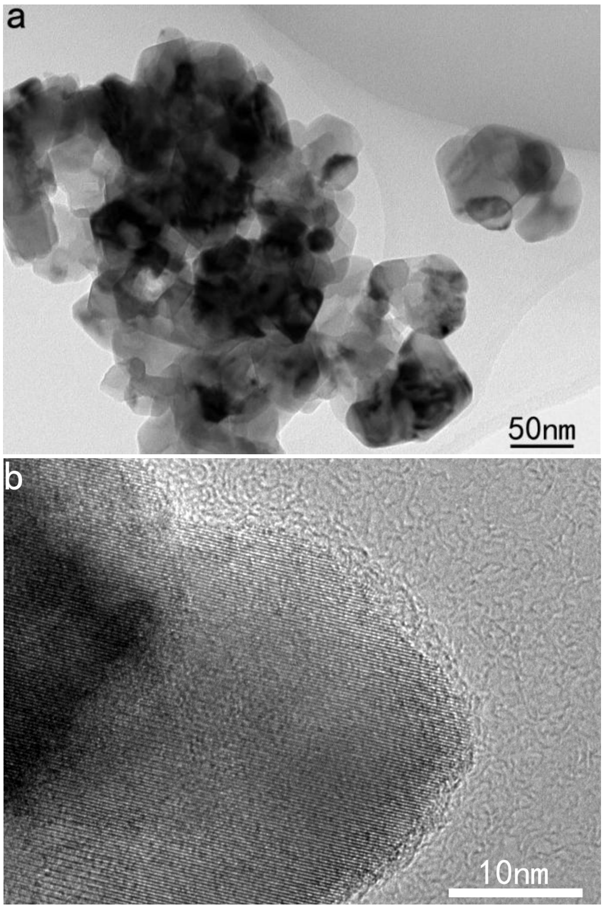

Supplementary Figure 10. TEM (a) and HRTEM (b) images of the crystalline δ−MoN irregular nanoparticles.

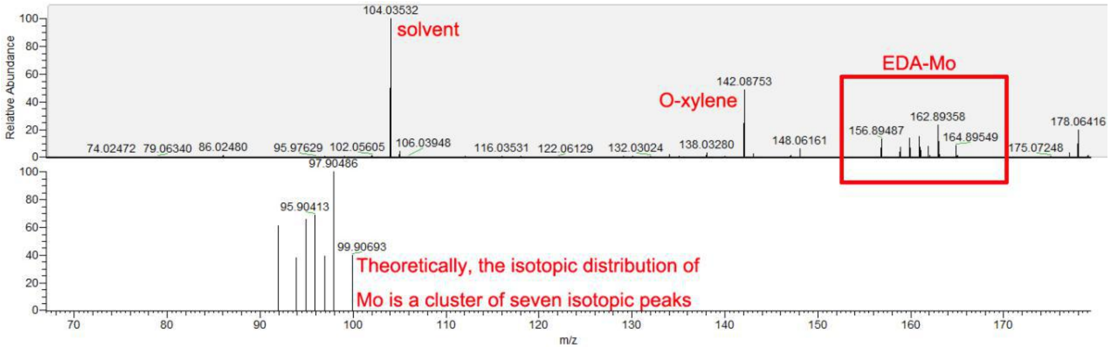

Supplementary Figure 11. High resolution mass spectrometry of the δ−MoN nanosheets. Theoretically, the isotopic distribution of Mo is a cluster of seven isotopic peaks. In the vicinity of 162.89358, after amplification, we can see that the measured peak is exactly the same as the theoretical isotopic distribution of Mo, indicating the formation of EDA-Mo.

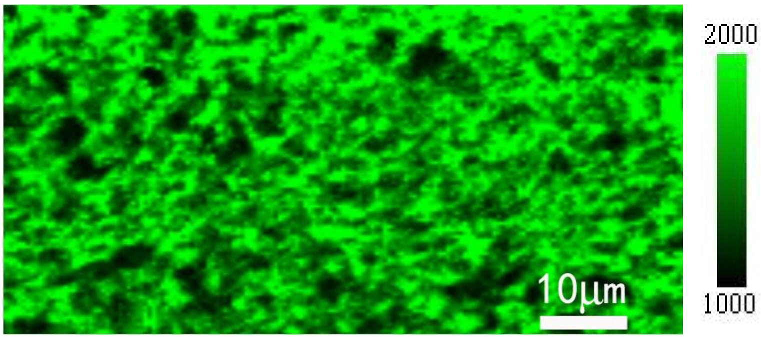

Supplementary Figure 12. SERS imaging shows that a large amount of EDA molecules are adsorbed on the surface of these δ–MoN nanosheets.

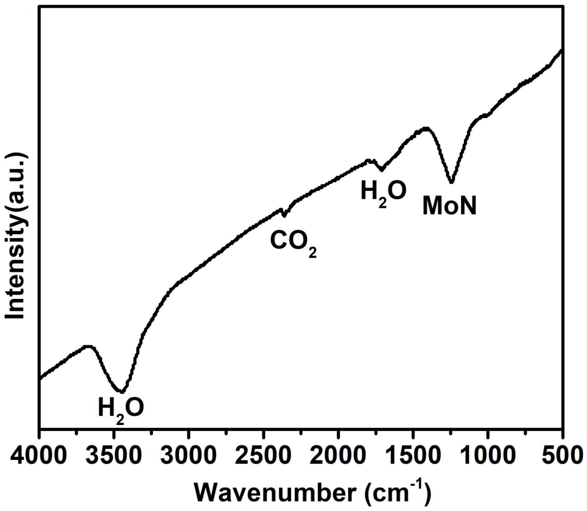

Supplementary Figure 13. FTIR spectrum of the MoN nanosheets after washing (ethanol, DMF and water) and freeze-drying.

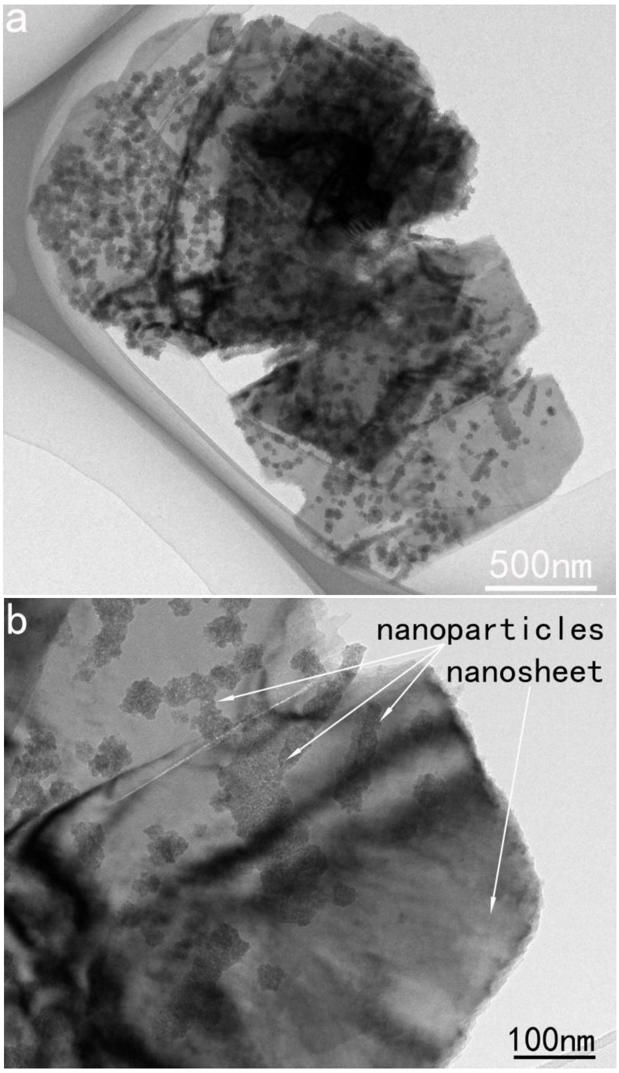

Supplementary Figure 14. Low-magnification and high-magnification TEM images of the δ−MoN product prepared at 350 °C.

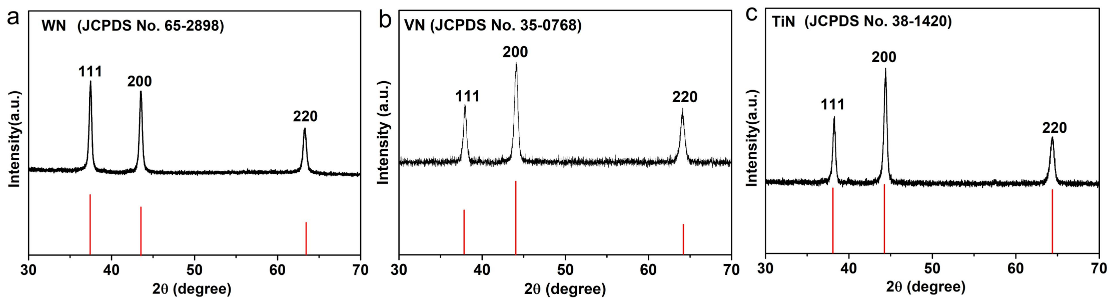

Supplementary Figure 15. XRD patterns of the WN, VN, and TiN nanosheets prepared by the low-temperature solution method, which demonstrates the generality of this method.

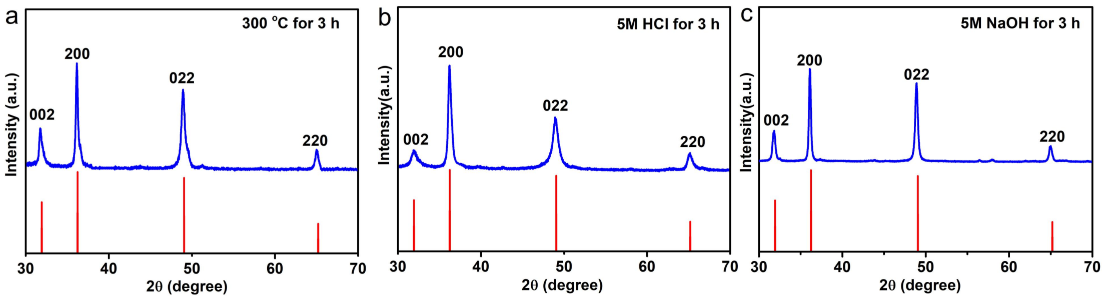

Supplementary Figure 16. XRD patterns of the δ–MoN nanosheets after (a) heating, (b) corrosion, and (c) irradiation, these almost invariable patterns prove the high stability of δ–MoN nanosheets.

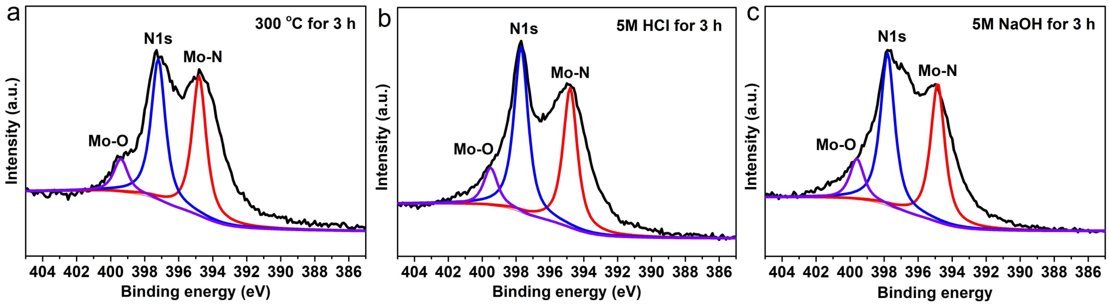

Supplementary Figure 17. XPS spectra of the δ–MoN nanosheets after (a) heating, (b) corrosion, and (c) irradiation, these almost invariable spectra further prove the high stability of δ–MoN nanosheets.

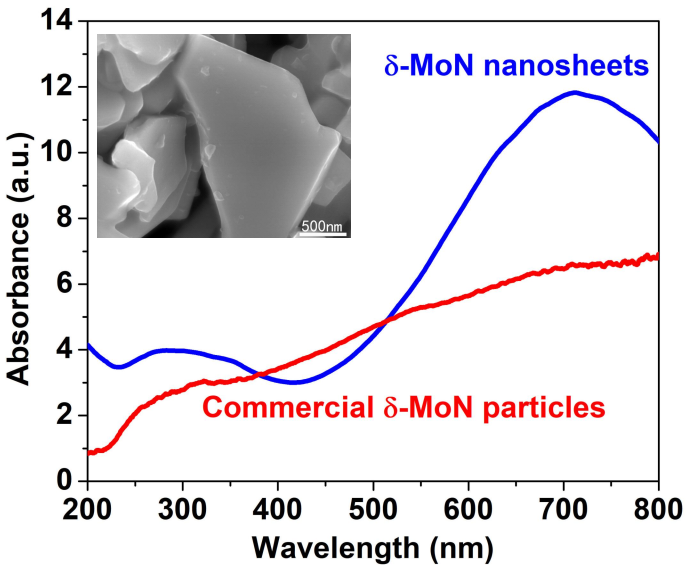

Supplementary Figure 18. UV−vis absorption spectra of the δ−MoN nanosheets and commercial δ-MoN powder, inset: SEM image of the commercial δ-MoN powder.

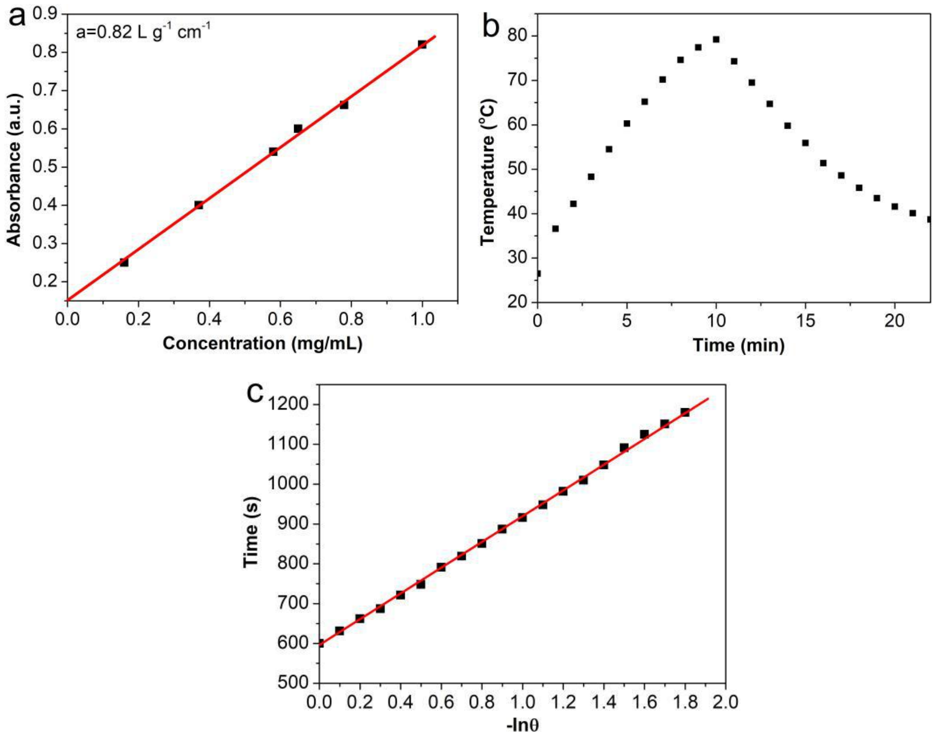

Supplementary Figure 19. (a) The linearly fitted plots of absorbance versus concentration of MoN nanosheet aqueous suspension at 633 nm. (b) Photothermal effect of aqueous dispersion of MoN nanosheets (1 mg/mL) under irradiation with the laser (633 nm, 1 W/cm^2) for one on/off cycle. (c) Time constant for heat transfer from the system is determined to be τs= 363.25 s by applying the linear time data from the cooling period (after 600 s) versus negative natural logarithm of driving force temperature, which is obtained from the cooling stage of (b).

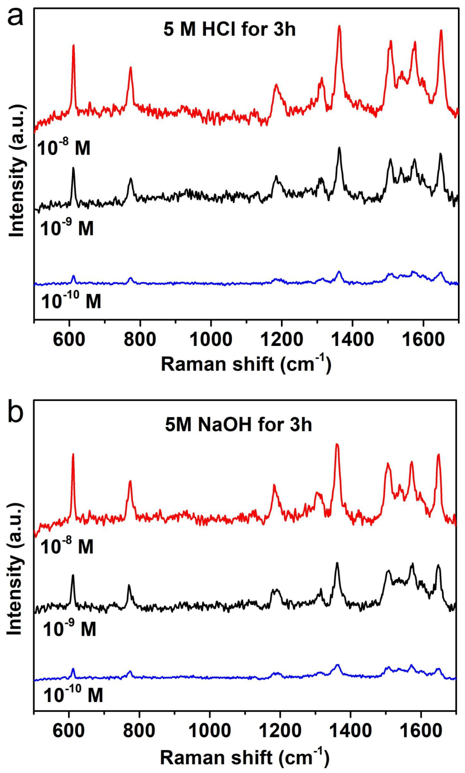

Supplementary Figure 20. Raman spectra of R6G recorded on the δ–MoN nanosheet substrate after strong acid (a) and strong alkali (b) corrosion, which proves its high corrosion resistance.

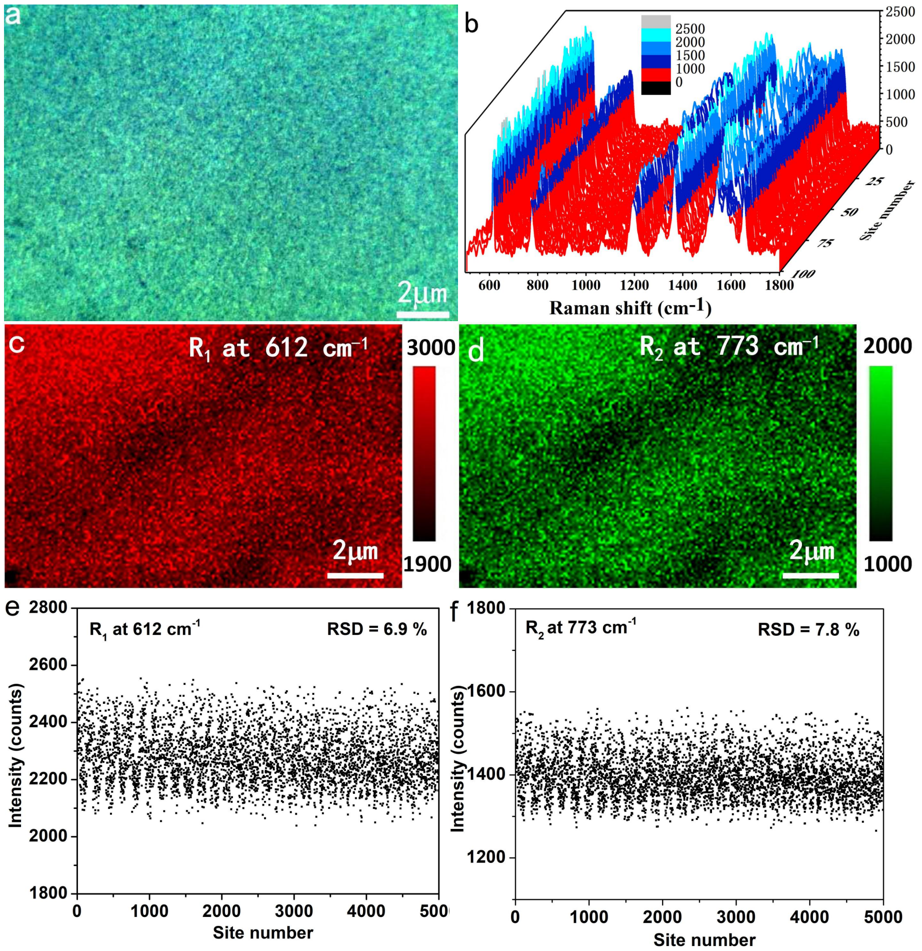

Supplementary Figure 21. Determination of the signal reproducibility and uniformity of the δ–MoN substrate. (a) Optical photograph of the substrate covered with δ–MoN nanosheets. (b) SERS signals collected from 100 randomly selected points on the substrate. (c-d) The SERS mapping and signal intensities at 612 cm^{-1} of 10^{-7} M R6G in the region shown in (a). (e-f) The SERS mapping and signal intensities at 773 cm^{-1} of 10^{-7} M R6G in the region shown in (a).

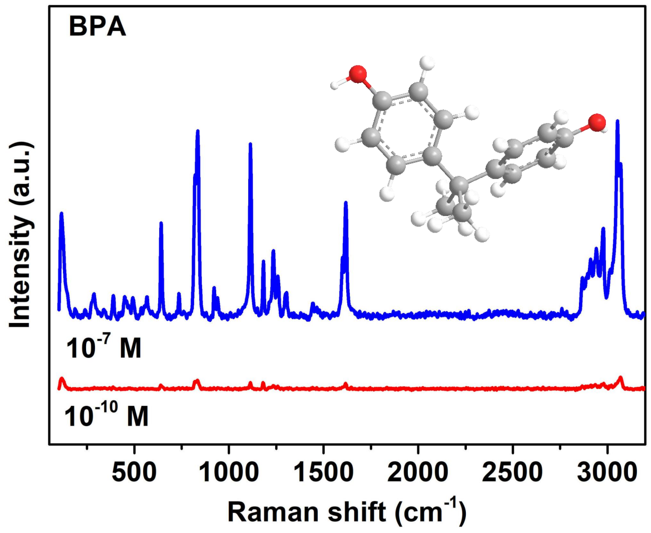

Supplementary Figure 22. Raman spectra of Bisphenol A (BPA) obtained in the flexible δ−MoN nanosheet substrate.

Supplementary Table 1: Some of the previously reported resistivity for non-metallic nanomaterials

**Component**	**Structure**	**Resistivity (Ω cm)**	**Synthesis condition**	**Reference**
δ-MoN	nanosheet	~2.5×10^{-4}	650 ºC	X. Xiao et al.[6]
δ-MoN	porous structure	~1.1×10^{-3}	650 ºC	Y. Yi et al.[7]
δ-MoN	film supported by Al_2O_3	~1.0×10^{-5}	950 ºC	H. M. Luo et al.[8]
δ-MoN	film supported by $SrTiO_3$	~2.0×10^{-5}	950 ºC	Y. Y. Zhang et al.[9]
$LaCoO_3$	film on $SrTiO_3$	~1.0	950 ºC	F. Rivadulla et al.[10]
$SrTiN_2$	Film on $LaAlO_3$	~4.7×10^{-4}	1000 ºC	M. Luo et al.[11]
$NiBaZr_{0.8}Y_{0.2}O_3$	nanopillars	~6.1×10^{-5}	1300 ºC	Q. Su et al.[12]
TiC	film supported by Al_2O_3	~3.7×10^{-4}	1000 ºC	G. F. Zou et al.[13]
WO_2	nanodendrites	~4.9×10^{-3}	180 ºC	Y. T. Ye et al.[14]
MoO_2	nanosheets	~6.2×10^{-3}	180 ºC	Q. Zhang et al.[15]
δ-MoN	nanosheets	~1.3×10^{-4}	270 ºC	This work

Supplementary References

(1) Kresse, G.; Furthmüller, J. Efficiency of ab-initio total energy calculations for metals and semiconductors using a plane-wave basis set. *Comp. Mater. Sci.* **6**, 15-50 (1996).

(2) Kresse, G.; Furthmüller, J. Efficient iterative schemes for ab initio total-energy calculations using a plane-wave basis set. *Phys. Rev. B* **54**, 11169-11186 (1996).

(3) Perdew, J.; P.; Burke, K.; Ernzerhof, M. Generalized gradient approximation made simple. *Phys. Rev. Lett.* **77**, 3865-3868 (1996).

(4) Kresse, G.; Joubert, D. From ultrasoft pseudopotentials to the projector augmented-wave method. *Phys. Rev. B* **59**, 1758-1775 (1999).

(5) Monkhorst, H.; J.; Pack, J. D. Special points for Brillouin-zone integrations. *Physical Review B* **13**, 5188-5192 (1976).

(6) Xiao, X. et al. Salt-templated synthesis of 2d metallic MoN and other nitrides. *ACS Nano* **11,** 2180-2186 (2017).

(7) Yi, Y. et al. Biotemplated synthesis of transition metal nitride architectures for flexible printed circuits and wearable energy storages. *Adv. Funct. Mater.* **28,** 1805510 (2018).

(8) Luo, H. M. et al. Controlling Crystal Structure and Oxidation State in Molybdenum Nitrides through Epitaxial Stabilization. *J. Phys. Chem. C* **115**, 17880–17883 (2011).

(9) Yingying Zhang et al. Epitaxial superconducting δ-MoN films grown by a chemical solution method. J. Am. Chem. Soc. **133**, 20735-20737 (2011).

(10) Rivadulla, F.; Bi, Z. X.; Bauer, E.; Murias, B. R.; Vila-Fungueirino, J. M. & Jia, Q. X. Strain-Induced Ferromagnetism and Magnetoresistance in Epitaxial Thin Films of $LaCoO_3$ Prepared by Polymer-Assisted Deposition. *Chem. Mater.* **25**, 55-58 (2013).

(11) Luo, H. M. et al. Epitaxial Ternary Nitride Thin Films Prepared by a Chemical Solution Method. *J. Am. Chem. Soc.* **130**, 15224–15225 (2008).

(12) Su, Q. et al. Self-Assembled Magnetic Metallic Nanopillars in Ceramic Matrix with Anisotropic Magnetic and Electrical Transport Properties. *ACS Appl. Mater. Interfaces* **8**, 20283-20291 (2016).

(13) Zou, G. F. et al. Chemical Solution Deposition of Epitaxial Carbide Films. *J. Am. Chem. Soc*. **132**, 2516–2517 (2010).

(14) Ye, Y. T. et al. A quasi-metallic tungsten oxide nanodendrites with high stability for surface enhanced Raman scattering, *Cell Re. Phy. Sci.* **1**, 100031 (2020).

(15) Zhang, Q. Q. et al. A metallic molybdenum dioxide with high stability for surface enhanced Raman spectroscopy. *Nat. Commun.* **8**, 14903 (2017).
