# Supplementary material for: Low temperature synthesis of plasmonic molybdenum nitride nanosheets for surface enhanced Raman scattering
Source: Nat Commun. 2020 Aug 4;11:3889. doi: 10.1038/s41467-020-17628-0 (PMC7403429; doi:10.1038/s41467-020-17628-0)
Supplement: Supplementary file 2 — Peer Review File [file 41467_2020_17628_MOESM2_ESM.docx]

Peer Review Information

**Journal:** Nature Communications

**Manuscript title:** Low temperature synthesis of plasmonic molybdenum nitride nanosheets for surface enhanced Raman scattering

**Corresponding author name(s):** Prof Guangcheng Xi

**Editorial notes:**

**Reviewer comments & decisions:**

**Reviewer comments, first version:**

Reviewer #1 (Remarks to the Author):

This manuscript presents a novel non-aqueous solvothermal process to synthesize crystalline MoN nanosheets using chemically active MoCl3 and Li3N as precursors, and further demonstrates its initial application in semiconductor SERS. Though the results are interesting, I would suggest a major revision with the comments noted below:
1. The synthesis involves precursors Li3N and EDA. This raises a few issues: a) The authors discuss in later sections that the as-obtained MoN has LSPR activity and is metallic, which is attributed to the formation of Mo-Mo bonds. However the authors overlooked the possibility of Li-doping in the MoN unit cell. It is known that doping of metallic ions in semiconductors raises the level of metallicity and introduces electrons into the conduction band. While the authors have provided EDS analysis of the elements in the material, I feel this is insufficient as Li is usually difficult to detect using EDS. Therefore it would be rigorous to provide further evidence that the as-obtained MoN is free of Li, or that the LSPR-related properties do not arise from Li. b) As the authors point out that the Raman signals of EDA can be detected on the as-obtained MoN, it is worth discussing how EDA is to be removed in the synthesis section, as otherwise this will cause signal interference in the following SERS analysis discussions.
2. There is a mistake regarding Figure 6d. The corresponding text reads “the center of the absorption is at 708.5 nm”, however in the figure the absorption peak is marked at 642 nm. This should be corrected.
3. Regarding Figure 7c which is a Raman mapping image, the original CCD image should accompany the mapping image, as an inset or adjacent figure.
4. In my opinion, molybdenum nitride (δ-MoN) doesn't belong in the MXene family according to the definition of MXene.


Reviewer #2 (Remarks to the Author):

The authors report in this manuscript the synthesis of delta-MoN ultra-thin nanosheets with high crystallinity at relatively mild conditions of 270 °C and 12 atm., and the evaluation of the properties of these nanosheets. They found that nanosheets show strong surface plasmon resonance effect, high conductivity, excellent thermal and chemical stability and a high photothermal conversion efficiency (~61.1%). These interesting results indicate that delta-MoN nanosheets could be a promising candidate material for a variety of applications, and deserve publication in the journal. I would suggest, however, the authors consider the follows before this manuscript might be acceptable for publication.

(1) Figure 7c. one sees that the Raman mapping resolution is less than absolutely 50 nm. It is better to state clearly the detailed measurement informations for readers to follow.

(2) Page 17, the laser power in 5 mW, while in page 22, it is said 0.5 mW. Is that a typo?

(3) SERS enhancement factors (EFs) has a clear definition and is a characteristic property of s substrate. Thus, the EF labelled in Figure 7e, may not be appropriate.

(4) Page 11. “SERS imaging” (Figure S10) was used to detect the distribution of EDA molecule. The color represents the signal strength, so it should have a color bar of Figure S10. Without the color bar, the difference between the green and black area is very confusing. Besides, is there a typo of the “Figure 10” in SI?

(5 Figure 5i, what is the laser spot size? It is better to point out clearly the irradiated area in the TEM image.

(6) It is suggest the authors make a survey in the introduction part to state clearly why they prefer develop delta-MoN nanosheets into SERS substrate in comparison with those well developed metallic substrates and/or nitride substrates.

**Author rebuttal, first version:**

**Reviewer 1**

1. The synthesis involves precursors Li3N and EDA. This raises a few issues: a) The authors discuss in later sections that the as-obtained MoN has LSPR activity and is metallic, which is attributed to the formation of Mo-Mo bonds. However the authors overlooked the possibility of Li-doping in the MoN unit cell. It is known that doping of metallic ions in semiconductors raises the level of metallicity and introduces electrons into the conduction band. While the authors have provided EDS analysis of the elements in the material, I feel this is insufficient as Li is usually difficult to detect using EDS. Therefore it would be rigorous to provide further evidence that the as-obtained MoN is free of Li, or that the LSPR-related properties do not arise from Li. b) As the authors point out that the Raman signals of EDA can be detected on the as-obtained MoN, it is worth discussing how EDA is to be removed in the synthesis section, as otherwise this will cause signal interference in the following SERS analysis discussions.

**Reply:** we are very grateful to the reviewer for his (her) suggestions. Let's first answer the question about whether Li exists. In order to more accurately measure whether Li is present in the MoN nanosheets, we adopted high-resolution ICP-MS. ICP-MS is very accurate to metal elements. By analyzing the results, the MoN nanosheets do not contain Li. Therefore, the excellent SERS properties can indeed be attributed to the metallicity and ultrathin nanostructures of MoN itself. Relative discussions have been added into the revised paper (please see page 8, yellow marked part). Next, we answer questions about EDA. Although the EDA molecules can be preferentially adsorbed on the MoN (101) crystal plane, the EDA molecules can be washed away with a suitable organic solvent, such as DMF. In fact, before doing the SERS experiments, the MoN nanosheets have been washed with ethanol, DMF, and water multiple times in order to ensure that almost no EDA remains. We have already described the washing details in the experimental part (please see page 21).

2. There is a mistake regarding Figure 6d. The corresponding text reads “the center of the absorption is at 708.5 nm”, however in the figure the absorption peak is marked at 642 nm. This should be corrected.

**Reply:** Thanks to the reviewers for pointing out our negligence, this error has been corrected (please see page 16).

3. Regarding Figure 7c which is a Raman mapping image, the original CCD image should accompany the mapping image, as an inset or adjacent figure.

**Reply:** we are very grateful to the reviewer for his (her) suggestions. According this suggestion, the original CCD image of this Raman mapping image has been inserted to Figure 7c (please see page 18).

4. In my opinion, molybdenum nitride (δ-MoN) doesn't belong in the MXene family according to the definition of MXene.

**Reply:** we thank the reviewer for sharing his opinion. We carefully checked the whole paper and found that we did not call these MoN nanosheets MXene. We just quoted some reports about MXene in the literature review. Throughout this article, we describe the products as ultrathin nanosheets. Despite this, we would like to thank the reviewers for their selfless sharing.

**Reviewer 2**

(1) Figure 7c. one sees that the Raman mapping resolution is less than absolutely 50 nm. It is better to state clearly the detailed measurement information for readers to follow.

**Reply:** we are very grateful to the reviewer for his (her) suggestions. The reason for this high resolution is due to the advanced equipment. The confocal micro Raman spectrometer we use is “inVia Qontor”, which is the most advanced Raman spectrometer developed by Renishaw. Its spectral resolution reaches FWHM 0.34nm at 585nm, and especially it has a powerful Raman imaging function. According to the suggestion of the reviewer, we have described the Raman imaging experimental procedure in detail in the method (please see page 22).

(2) Page 17, the laser power in 5 mW, while in page 22, it is said 0.5 mW. Is that a typo?

**Reply:** thank you very much for the reviewer for pointing out our carelessness. “5 mW” has been changed into “0.5 mW”.

(3) SERS enhancement factors (EFs) has a clear definition and is a characteristic property of substrate. Thus, the EF labelled in Figure 7e, may not be appropriate.

**Reply:** we thank the reviewer for sharing his opinion. In fact, the Figure form we used is a form widely used to evaluate Raman EF of semiconductors, such as W_18_O_49_ (Nat. Commun. 6, 7800, 2015.), Cu_2_O (Adv. Mater. 2017, 29, 1604797), ZnO (Angew. Chem. Int. Ed. 56, 2017, 9851) Furthermore, this method is also used in previous articles published by our group, such as Nat. Commun. 2017, 8, 14903. Therefore, we have reason to believe that the current EF calculation method is reasonable.
(4) Page 11. “SERS imaging” (Figure S10) was used to detect the distribution of EDA molecule. The color represents the signal strength, so it should have a color bar of Figure S10. Without the color bar, the difference between the green and black area is very confusing. Besides, is there a typo of the “Figure 10” in SI?

**Reply:** we are very grateful to the reviewer for his (her) suggestions. Accordong this suggestion, we have added a color bar into the Figure S10. At the same time, the “Figure 10” has been corrected to “Figure S10”.

(5) Figure 5i, what is the laser spot size? It is better to point out clearly the irradiated area in the TEM image.

**Reply:** we are very grateful to the reviewer for his (her) suggestions. The laser spot diameter is about 700 μm. The TEM image shown in Figure 5i is a small part of the area under laser irradiation. The relative data has been added into the revised paper (please see page 15).

(6) It is suggest the authors make a survey in the introduction part to state clearly why they prefer develop delta-MoN nanosheets into SERS substrate in comparison with those well developed metallic substrates and/or nitride substrates.

**Reply:** This is a good suggestion and we are very grateful to the reviewer for this suggestion. According to this suggestion, we have added a survey in the introduction. Relative contents please see page 4.

**PS:** In accordance with the requirements for the number of words in the title and abstract, we have revised the content of the title and abstract, please check whether it is appropriate.

**Reviewer comments, second version:**

Reviewer #1 (Remarks to the Author):

This work reports on synthesis of metal nitride nanosheets directed to surface enhanced Raman spectroscopy uses. However, the general way for preparing nanosheets of this kind or employing Li3N as nitrogen source for producing transition metal nitride has been ever reported (Chemical Science, 2014, 5, 4615, Coordination Chemistry Reviews 2013, 257, 2063 and references therein); from the aspect of semiconductor-based SERS substrate, there are already a large number of reports in the literature, and no significant advance to this field can be recognized in this work. In addition, there are many unreliable descriptions. Considering these points, I cannot recommend to publish in Nature Communications. Other specific comments are as follows:

1. For the possible effect of EDA, although the authors added additional statement of EDA washing procedures and stated that the surface was EDA–free (“no EDA remains”), but no any evidence was given. EDA works as directing agent and binds with specific surface of the nitride nanosheets, and I do not believe all EDA can be removed.
2. For the answer of the forth question, the authors responded that “We carefully checked the whole paper and found that we did not call these MoN nanosheets MXene. We just quoted some reports about MXene in the literature review.”, but lines 63-64 say “Gogotsi et al. prepared δ−MoN MXene and arrayed nanocrystals at 650 °C ….”. Although the description is restricted to the literature review section, still the MoN is not MXene. Strict description and explanation is an essential requirement in a scientific paper.
3. The indexing in Figure 2e is obviously wrong, as the indexed lattice planes do not lie in the indicated zone axis of [101]. I recommend the authors check the indexing throughout the manuscript.
4. Line 148, the authors give thickness of 2-5 nm from SEM characterization, which is not convincing enough. The information on nanosheet thickness should be given by providing AFM evidences. In addition, in Figure 2g the authors marked 1L 2L 3L 4L 5L by discerning the layer-by-layer extruding part, but it should be noted that each extruding part may be a stack of several layers. The thickness difference in Figure 2g for the marked 3L to 4L and 4L to 5L is definitely more than one layer. Again, the number of layers should be given by AFM characterizations.
5. In order to prove the elemental ratio of Mo:N, it is essential to characterize EDS mapping of a larger area.
6. The conductivity derived from the current-voltage measurement is suggested to be given; a comparison with other typical materials and MoN of other nanostructures may be meaningful.
7. The occurrence of LSPR phenomenon was reasoned to be resulted from high electrical conductivity. It is well known the delta-MoN of many forms, not limited to nanosheets, possesses excellent conductivity even superconductivity at certain temperature, then it is reasonable to expect that those MoN also have LSPR characteristics. Is this true? If yes, the authors should provide the importance of making thin nanosheets of MoN.
8. The surfaces of the MoN nanosheets were superhydrophobic, proved by contract angle measurement, while the molecules adopted for Raman measurement were in aqueous solution. How to avoid aggregation of molecules and ensure uniform spreading of the probe molecules?


Reviewer #2 (Remarks to the Author):

I would suggest to accept the manuscript in the revised form to publication.

**Author rebuttal, second version:**

**Reviewer-1's general comments**

This work reports on synthesis of metal nitride nanosheets directed to surface enhanced Raman spectroscopy uses. However, the general way for preparing nanosheets of this kind or employing Li3N as nitrogen source for producing transition metal nitride has been ever reported (Chemical Science, 2014, 5, 4615, Coordination Chemistry Reviews 2013, 257, 2063 and references therein); from the aspect of semiconductor-based SERS substrate, there are already a large number of reports in the literature, and no significant advance to this field can be recognized in this work. In addition, there are many unreliable descriptions. Considering these points, I cannot recommend to publish in Nature Communications.

**Reply:** We are very grateful for the literature information provided by the reviewer. After carefully studying the two articles provided by the reviewer and other papers referenced in the two articles, we found that the methods described in these articles are very different from the method we reported.

(1) In the first article (Chemical Science, 2014, 5, 4615-4620), Prof. Xie et al. reported the metallic MoN nanosheets with atomic thickness as a highly efficient platinum-free electrocatalyst for hydrogen evolution reaction. The atomically-thin MoN nanosheets were fabricated by ***liquid exfoliation*** of the bulk precursor material in N-methyl-2-pyrrolidone (NMP). In their method, the preparation of the precursor requires high temperature (**650 ^o^C for 6 h**) and irritant ammonia. It should be said that this is a conventional two-step liquid-phase exfoliation method. Therefore, this method reported by Xie et al. is completely different from our one-step low temperature solution growth method, and the entire preparation process does not involve Li3N. For ease of reference, the method reported by Xie et al. has been attached below (Chemical Science, 2014, 5, 4615):

**Synthesis of the MoN bulk material**

The MoN bulk material was synthesized by annealing a chemically homogeneous molecular precursor under ammonia atmosphere. The Mo-containing precursor was prepared by a method reported previously.^42^ In detail, 3.5 g of (NH4)4Mo7O24·4H2O (in 50 mL distilled water) and 6 g of hexamethylenteramine (HMT, in 50 mL distilled water) were mechanically mixed, and colorless crystals (ammonium-HMT molybdate mixed salt) was formed after standing still for several hours at ambient conditions. The solid precursor was then collected by decanting the supernatant, washed with a small amount of water, and dried in air under room temperature. The mixed salt precursor was then annealed at **650 °C** (with heating rate of 10 °C min^-1^) for 6 hours under flowing ammonia (50 mL min^-1^) inside a fused quartz tube (Caution: Ammonia is a corrosive, toxic gas which must be used in a properly ventilated area.), and finally the dark grey MoN bulk material was obtained. **Synthesis of the atomically-thin MoN nanosheets by liquid exfoliation**

The atomically-thin MoN nanosheets were obtained by ***liquid exfoliation*** of the as-synthesized MoN bulk material in Nmethyl-pyrrolidone (NMP). Typically, 50 mg of bulk MoN and 20 mL of NMP were loaded in a weighing bottle with a total capacity of 20 mL. After ultrasound treatment for about 8 hours to ensure the complete exfoliation, the resultant dispersion was centrifuged at 3000 rpm to remove the residual unexfoliated MoN particles, and the exfoliated atomically-thin MoN nanosheets were finally collected by high-speed centrifugation at 12000 rpm for 3 min and washed by ethanol for further characterizations.

(2) As for the second article (Coordination Chemistry Reviews 2013, 257, 2063-2072), this is a review about metal nitrides (entitled “Nitrogen-rich transition metal nitrides”). In the first paragraph on page 4 of this review, the author discusses the use of solid state metathesis reactions for the synthesis of metal nitrides. In this method, Li3N and Na3N are used as nitrogen sources. We have carefully read the references in this section ([54-58]: J. Chem. Soc. Chem. Commun. 1993 1095*;*Inorg. Chem. 1994, 33, 5693; Chem. Mater. 1995, 7, 1728; J. Chem. Soc. Dalton Trans. 1993, 2435; Polyhedron 1995, 14, 913), and we found that this solid-state reaction is

actually a high-temperature self-propagating reaction, and the temperature is as high as 900-1300 ^o^C. And the reaction process is extremely fast (basically finished in a few seconds), so it is difficult to control the morphology of the products. As a predictable result, these metal nitride products are irregular particles. Therefore, this solid-state high-temperature metathesis reaction is quite different from the mild solution method reported by us in terms of reaction environment, temperature, kinetics and product morphology.

**Reviewer-1's specific comments**

1. For the possible effect of EDA, although the authors added additional statement of EDA washing procedures and stated that the surface was EDA–free (“no EDA remains”), but no any evidence was given. EDA works as directing agent and binds with specific surface of the nitride nanosheets, and I do not believe all EDA can be removed.

**Reply:** We are very grateful to the reviewer for this question, and we are very sorry for our inadequate answers. According to the reviewer's suggestion, we carefully examined the surface composition of the MoN nanosheets after cleaning with solvents through various techniques. First, the FTIR spectrum characterization results show that the surface of the MoN nanosheets does not show the obvious presence of EDA after cleaning; second, the Raman spectrum detection of the MoN nanosheets shows that no Raman signal of EDA appears, only the Raman signal of MoN itself is detected. In addition to the cleaning effect of the solvents, we think that the final freeze-drying also plays a role in the removal of EDA molecules. Because freeze-drying is carried out under considerable negative pressure, this should be helpful for the volatilization of organic molecules. These results suggest that after sufficient cleaning, the MoN nanosheets are indeed very clean and will not affect future SERS detection. Of course, we also believe that indeed, as the reviewers said, there may be a very small amount of EDA molecules on the surface of the nanosheets, but this will hardly affect the SERS detection. Related discussions and supplementary results have been added to the revision **(please see page 12 and Supplementary Figure 13**). Thanks again for the reviewer's suggestions, which is very helpful for

improving the quality of the article.

**Page 12:** It should be noted that these adsorbed EDA molecules can be washed away in a suitable solvent. FTIR spectrum showed that no EDA signal was detected on the surface of MoN nanosheets after being washed with DMF and ethanol (Supplementary Figure 13). At the same time, the Raman spectrum detection of the MoN nanosheets shows that no Raman signal of EDA appears, only the Raman signal of MoN itself is detected (Supplementary Figure 1).

**Supplementary Figure 13.** FTIR spectrum of the MoN nanosheets after washing (ethanol, DMF and water) and freeze-drying.

2. For the answer of the forth question, the authors responded that “We carefully checked the whole paper and found that we did not call these MoN nanosheets MXene. We just quoted some reports about MXene in the literature review.”, but lines 63-64 say “Gogotsi et al. prepared δ−MoN MXene and arrayed nanocrystals at 650 °C ....”. Although the description is restricted to the literature review section, still the MoN is not MXene. Strict description and explanation is an essential requirement in a scientific paper.

**Reply:** We are very grateful for the reviewer to point out that our description is not rigorous. Based on this suggestion, we rewrote this description (**please see page 3**) according to the original description: “Gogotsi et al. prepared δ−MoN *nanosheets* and arrayed nanocrystals at 650 °C.”

3. The indexing in Figure 2e is obviously wrong, as the indexed lattice planes do not lie in the indicated zone axis of [101]. I recommend the authors check the indexing throughout the manuscript.

**Reply:** We are very grateful for the error pointed out by the reviewers, and I am very sorry for this error. I checked these SAED pattern (Figure 2e) and HRTEM image (Figure 2h) and found that the interpretation was indeed wrong. The main reason for this error is that my students erroneously measured the interplanar spacing, and I sincerely apologize for my negligence. In fact, the (112) crystal plane should be indexed as the (020) crystal plane, and the crystal axis should be [00-1] instead of [101]. In the revision, I have corrected the calibration of these patterns and crystal planes in Figure 2e, Figure 2h and Figure 3b-d according to the knowledge of crystallography. Thanks again for the reviewer's comment, which is very helpful for improving the quality of the article.

Figures 2e and 2h after correction:

Figures 3b-d after correction:

4. Line 148, the authors give thickness of 2-5 nm from SEM characterization, which is not convincing enough. The information on nanosheet thickness should be given by providing AFM evidences. In addition, in Figure 2g the authors marked 1L 2L 3L 4L 5L by discerning the layer-by-layer extruding part, but it should be noted that each extruding part may be a stack of several layers. The thickness difference in Figure 2g for the marked 3L to 4L and 4L to 5L is definitely more than one layer. Again, the number of layers should be given by AFM characterizations.

**Reply:** This is a very good suggestion and we have made AFM experiments according to this suggestion. A series of AFM pictures show that the thickness of these MoN nanosheets is indeed the same as that observed by the electron microscope. **Related descriptions (page 7) and images (Supplementary Figure 4)** were added to the revision. We agree with the reviewers' views on Figure 2g. We modified the description of this image to attribute this phenomenon to growth steps.

**Supplementary Figure 4.** The thicknesses of -MoN nanosheets was determined

using AFM. (a) AFM images of the MoN nanosheets deposited on Si. (b) AFM height profiles measured along the white line in (a).

5. In order to prove the elemental ratio of Mo:N, it is essential to characterize EDS mapping of a larger area.

**Reply:** This is a very good suggestion and we have made supplementary experiments according to this suggestion. The EDS spectrum obtained in larger area also shows that the ratio of molybdenum to nitrogen is 1.07. Considering that EDS is a semi-quantitative measurement method, these results indicate that molybdenum/nitrogen atom ratio is very close to 1/1. **Relative discussion please page 8 and Supplementary Figure 7**.

**Supplementary Figure 7.** EDS spectrum obtained in a large area MoN nanosheets.

6. The conductivity derived from the current-voltage measurement is suggested to be given; a comparison with other typical materials and MoN of other nanostructures may be meaningful.

**Reply:** This is a very good suggestion. After calculation, the resistivity of the MoN nanosheet film derived from the current-voltage measurement is ∼ 1.29 × 10^-4^ Ωcm. The relative content has been added into the revised manuscript (please see page 16). According to the suggestion of the reviewer, we also compared the resistivity of several non-metallic nanomaterials and found that the conductivity of the MoN nanosheets is relatively good (**please see Supplementary Table 1**).

**Supplementary Table 1:** Some of the previously reported resistivity for non-metallic nanomaterials

| **Component** | **Structure** | **Resistivity  (**Ω cm) | **Synthesis  condition** | **Reference** |
| --- | --- | --- | --- | --- |
| -MoN | nanosheet | -2.5 × 10^-4^ | 650 o_C_ | X. Xiao et al.^1^ |
| -MoN | porous  structure | -1.1 × 10^-3^ | 650 o_C_ | Y. Yi et al.^2^ |
| -MoN | film  supported by  Al2O3 | -1.0 × 10^-5^ | 950 o_C_ | M. Luo et al.^3^ |
| Mo2N | film  supported by  SrTiO3 | -2.0 × 10^-5^ | 950 o_C_ | H. M. Luo et al.^3^ |
| LaCoO3 | film on  SrTiO3 | -1.0 | 950 o_C_ | F. Rivadulla et al.^4^ |
| SrTiN2 | Film on  LaAlO3 | -4.7 × 10^-4^ | 1000 o_C_ | M. Luo et al.^5^ |
| NiBaZr0.8Y0.2O3 | nanopillars | -6.1 × 10^-5^ | 1300 o_C_ | Q. Su et al.^6^ |
| TiC | film  supported by  Al2O3 | -3.7 × 10^-4^ | 1000 o_C_ | G. F. Zou et al.^7^ |
| WO2 | nanodendrites | -4.9 X 10^-3^ | 180 o_C_ | Y. T. Ye et al.^8^ |
| MoO2 | nanosheets | -6.2 X 10^-3^ | 180 o_C_ | Q. Zhang et al.^9^ |
| -MoN | nanosheets | -1.3X 10^-4^ | 270 o_C_ | This work |

7. The occurrence of LSPR phenomenon was reasoned to be resulted from high electrical conductivity. It is well known the delta-MoN of many forms, not limited to nanosheets, possesses excellent conductivity even superconductivity at certain temperature, then it is reasonable to expect that those MoN also have LSPR characteristics. Is this true? If yes, the authors should provide the importance of

making thin nanosheets of MoN.

**Reply:** Thanks to the reviewer for providing us such a very good suggestion. Indeed, as the reviewers said, the conductivity of ö-MoN is good, it may show the LSPR effect. In general, materials that exhibit a strong LSPR effect have better conductivity, but conversely, materials with good conductivity do not necessarily exhibit excellent LSPR effects. Just like gold and platinum, they are both good conductors, but gold is a good LSPR material, while platinum is not. Even if it is all a material such as gold, different nanostructures will profoundly affect the strength and location of their LSPR. As a comparison, we measured the UV-Vis absorption of commercially available ö-MoN particles (micron level). The results show that although these irregular micron-sized particles show a certain absorption in the visible range, their intensity is much lower than that of these ö-MoN nanosheets, and no absorption peak is formed. These results indicate that the morphology and size of ö-MoN have a great influence on its LSPR effect. Although the exact structure-effect relationship cannot be explored clearly at present, we believe this will be a very worthy research topic. We have added the results of the comparison experiment to the revised manuscript, and emphasized the significance of the structure for the LSPR effect of ö-MoN nanosheets based on the reviewer’s suggestion (**please see page 16 and Supplementary Figure 18**).

**Supplementary Figure 18.** UV−vis absorption spectra of the ö-MoN nanosheets and commercial ö-MoN powder, inset: SEM image of the commercial ö-MoN powder.

8. The surfaces of the MoN nanosheets were superhydrophobic, proved by contract angle measurement, while the molecules adopted for Raman measurement were in aqueous solution. How to avoid aggregation of molecules and ensure uniform spreading of the probe molecules?

**Reply:** We are very grateful to the reviewers for asking this question. In our experiment, when the probe molecules are 2,5-DCP and BPA, because they are insoluble in water, the solvent we chose is ethanol (this part is not mentioned in the original experiment part, it has been added in the revision). The ethanol solution of these substances can be very uniformly distributed on the MoN substrate. This uniform distribution has been proven by Raman mapping (please see Supplementary Figure 15). When the probe molecule is R6G, the solvent is a mixture of water and a small amount of ethanol (sorry, we wrote it as an aqueous solution in the original experimental part due to carelessness). We have detailed the preparation process of the probe sample in the revision. Furthermore, we systematically tested and calculated the relative standard deviation (RSD) of the Raman signals of the probe molecules on the MoN substrate, and their values were between 6-8 %. **Relative content please see page 20-21 and Supplementary Figure 21**.

**Supplementary Figure 21.** Determination of the signal reproducibility and uniformity of the -MoN substrate. (a) Optical photograph of the substrate covered with -MoN nanosheets. (b) SERS signals collected from 100 randomly selected points on the substrate. (c-d) The SERS mapping and signal intensities at 612 cm^-1^ of 10^-7^ M R6G in the region shown in (a). (e-f) The SERS mapping and signal intensities at 773 cm^-1^ of 10^-7^ M R6G in the region shown in (a).

**Reviewer comments, third version:**

Reviewer #2 (Remarks to the Author):

I would like to suggest acceptance of this manuscript in the revised form for publiation.

**Author rebuttal, third version:**
